# Aging clocks based on accumulating stochastic variation

**DOI:** 10.1038/s43587-024-00619-x

**Published:** 2024-05-09

**Authors:** David H. Meyer, Björn Schumacher

**Affiliations:** 1 Institute for Genome Stability in Aging and Disease, University Hospital and University of Cologne, Cologne, Germany; 2 Cologne Excellence Cluster for Cellular Stress Responses in Aging-Associated Diseases (CECAD), Center for Molecular Medicine Cologne (CMMC), University of Cologne, Cologne, Germany

**Keywords:** Computational models, Ageing, Ageing

## Abstract

Aging clocks have provided one of the most important recent breakthroughs in the biology of aging, and may provide indicators for the effectiveness of interventions in the aging process and preventive treatments for age-related diseases. The reproducibility of accurate aging clocks has reinvigorated the debate on whether a programmed process underlies aging. Here we show that accumulating stochastic variation in purely simulated data is sufficient to build aging clocks, and that first-generation and second-generation aging clocks are compatible with the accumulation of stochastic variation in DNA methylation or transcriptomic data. We find that accumulating stochastic variation is sufficient to predict chronological and biological age, indicated by significant prediction differences in smoking, calorie restriction, heterochronic parabiosis and partial reprogramming. Although our simulations may not explicitly rule out a programmed aging process, our results suggest that stochastically accumulating changes in any set of data that have a ground state at age zero are sufficient for generating aging clocks.

## Abstract (web summary)

Meyer and Schumacher use simulations to show that accumulation of stochastic variation is sufficient to build clocks that can measure both chronological and biological age, sensitive to changes induced by smoking, calorie restriction, parabiosis and reprogramming.

## Main

Weismann’s 1881 proposition suggested an aging program to benefit species by freeing up resources from older individuals^1^. This hypothesis was later largely rejected^2–5^, for a range of reasons such as the circularity of the argument and the assumption of group selection. Evolutionary theories of aging realized the vanishing force of natural selection post-reproductively, notably stated in the disposable soma, mutation accumulation and antagonistic pleiotropy theories of aging^2,6^. Mutations that abruptly limit post-reproductive life are observed in semelparous species, whereas iteroparous species typically show a gradual functional decline because of insufficient maintenance and repair mechanisms, leading to stochastic damage accumulation with aging^7^. Progress on aging clocks has revived the idea of a potential aging program^8^, questioning whether aging is primarily a stochastic entropy-driven event, whether aging clocks could show a causal relationship^9,10^ and whether it involves programmatic aspects^11–16^. Intrinsic flaws in a software code of life^17^, an adaptive pathogen control program^11,18^ or developmental processes^13,15^ were suggested to cause aging. Age-dependent selective mortality may depend not only on remaining fertility, but also on intergenerational resource transfer, explaining a quantity–quality tradeoff, and potentially allowing a programmed process to affect aging^19^.

Epigenetic drift, observed during aging, was assigned to imperfect maintenance of epigenetic marks^20^, reducing methylation differences between genomic regions that are defined during development over time^21^. It has been proposed that age-coupled stochastic methylation changes are highly genome context specific^22^, and that an information-theoretic view of DNA methylation pattern explains the observed stochasticity in line with context-specific maintenance energy consumption^23^. Differential equations showed that CpG methylation sites can be modeled based on maintenance rates, defining CpG site-specific equilibria^24,25^. Horvath’s epigenetic clock was suggested to result from an imperfect epigenetic maintenance system (EMS)^26^ and increased DNA methylation entropy was observed in older individuals^27^. This stochastic epigenetic drift is conserved across species and attenuated upon caloric restriction^28^. Age-related variably methylated positions are reproducible, not driven by cell-type composition, linked to developmental and DNA damage response genes, enriched at polycomb repressed regions and associated with expression of polycomb repressive complex 2 (ref. ^29^). Moreover, ~30% of the mouse genome might be affected by age-related epigenetic disorder, which is enriched in the Petkovich clock^30^, and a clock using these biological disorder measurements could be built^31^.

To deepen the mechanistic understanding of epigenetic aging clocks, CpG sites from 12 clocks were deconstructed into distinct modules some of which might be driven by entropic alterations that regress to a methylation state of 0.5, whereas most modules change systematically with time^32^. Recently, it was demonstrated that initializing CpG values at either 0% or 100% could accurately predict the simulated age in single-cell simulations, irrespective of stochastic, coregulated or combined simulation. Starting every CpG site at 0% or 100%, they could either remain unchanged or regress toward 0.5 (ref. ^33^), suggesting that a single stochastic variable could track entropic aging^34^.

Here, we show that datasets that contain accumulating stochastic variation, and are normalized between 0 and 1, can be used to build an age predictor suggesting that any set of biological measurements could be used to build accurate aging clocks. The pace of predicted aging is primarily set by the degree of stochastic variation, where increased stochasticity accelerates, whereas reduced stochastic variation decelerates the predicted age. Predictions of a transcriptomic aging clock for *Caenorhabditis elegans* correlate significantly with the amount of added stochastic variation. The predictive results of a clock based on simulated transcriptomic data with accumulating stochastic variation significantly correlate with chronological age. Epigenetic aging clocks measure how much stochastic variation has accumulated, and the predictive results of a model trained on simulated data with accumulating stochastic variation correlate significantly with the chronological age of human DNA methylation samples. We validated and replicated our results on data from the Mammalian Methylation Consortium^35^, showing that a variety of mammalian species and interventions can be correctly predicted. We establish that the accumulation of stochastic variation is enabling the construction of pan-mammalian clocks, which are capable of detecting biological age deceleration and acceleration^15^, and the rejuvenation trajectory over a reprogramming time-course in human cells. Our analyses suggest that aging clocks could be based on any biological parameter with stochastic age-related alterations for precise measurements of aging, without the need for a deterministic process.

## Results

### Data-type independent predictions

**Extended Data Fig. 1:**
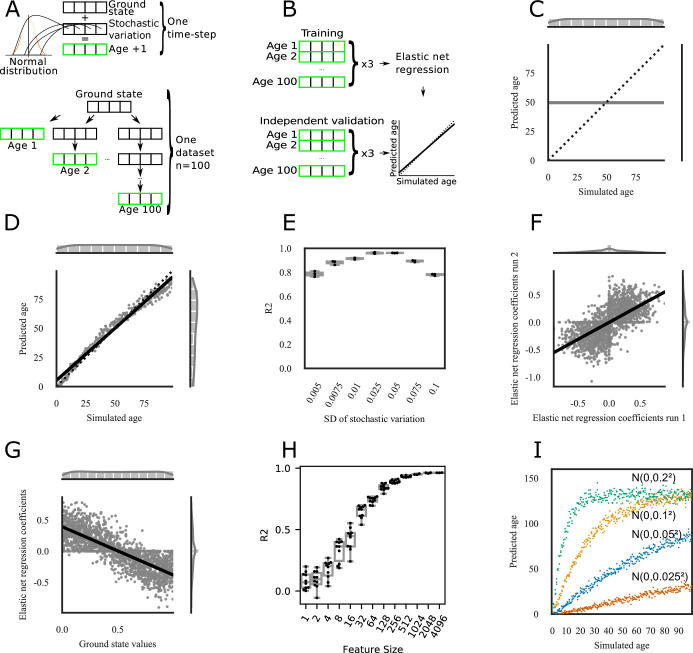
Normal-distributed stochastic variation accumulation simulations with value limits enable aging clock construction for simulated data. **a**) Sample generation explanation. One time-step is defined as the addition of one-time stochastic variation, that is random noise, to each feature of the ground state that is sampled from a normal distribution centered at 0 (Top). Samples with different simulated ages are generated starting from the same ground state, but independently from each other (Bottom). A sample of age 1 adds normal-distributed stochastic variation once to the ground state, a sample of age 2 twice independently, and so on. **b**) Model training and validation explanation. For training and validation 3 sets of independent samples are generated from the same ground state as explained in Extended Data Fig. 1a. 3 sets comprising the whole age-range, for example 1–100, are used as an input for an Elastic net regression to train a predictor that predicts the simulated age of a sample, that is how often stochastic variation was added to the ground state. The 3 independent datasets are used to validate the model and assess the accuracy. **c**) Unlimited stochastic variation does not allow for any prediction. All samples within the training and validation dataset started from the same ground state of 2000 uniformly randomly sampled features between 0 and 1. For every whole simulated age step from 1 to 100, normal-distributed stochastic variation sampled from *N*(µ = 0, *σ*^2^ = 0.05^2^) was added. n = 300 samples (3 independent samples per age step) were used for training of the Elastic net regression model to predict the simulated age, and n = 300 independent samples were used for validation. The x-axis shows the true simulated age, that is the number of times random stochastic variation was added to the ground state. The y-axis shows the prediction of the Elastic net regression model of the independent validation data (n = 300, 3 independent samples per time point). The sides show the distribution of the samples. **d**) Same as C), but after addition of stochastic variation the values were kept within the range of 0–1, for example values bigger to 1 were set to 1 (n = 300, 3 independent samples per time point). Limiting the values after stochastic variation application allows to build highly accurate predictors of the simulated age. **e**) The predictions of the independent validation data are robust to the stochastic variation distribution. The samples were simulated the same as in D) with different stochastic variation distributions (n = 300, 3 independent samples per time point). The x-axis shows the standard deviation of the normal distribution from which the stochastic variation was sampled, that is *N*(µ = 0, *σ*^2^ = 0.005^2^) has a narrow noise distribution with 99.7 % of the sampled data within the range [−0.015, 0.015], while *N*(µ = 0, *σ*^2^ = 0.01^2^) has a wide distribution with 99.7 % of the sampled data within the range [-0.3, 0.3]. The y-axis shows the R² value between the simulated age and the predicted age of the independent validation data (N = 3 independent repeats; each with n = 300, 3 samples per time point). Boxplots are shown with the center line depicting the median, the box limits the bottom, respective top quartiles, and the whiskers the 1.5x interquartile range. **f**) Independent Elastic net regression models are highly correlated if trained on samples starting from the same ground state (consisting of N = 2000 uniformly randomly sampled features between 0 and 1). The x-axis shows the coefficients of the Elastic net regression of D), and the y-axis shows the coefficients of an independent Elastic net regression on samples that started with the same ground state, but with independent stochastic variation application (trained on n = 300, 3 samples per time point). **g**) The prediction in D) is possible due to a regression to the mean. The x-axis shows the starting values of the 2000 features of the simulated ground state, the y-axis the Elastic net regression coefficients for the model in D) (trained on n = 300, 3 samples per time point). Features starting close to 0 have a positive coefficient, indicating an increase over the simulated time period, while features close to 1 have a negative coefficient, indicating a decrease. Features close to 0.5 are more sensitive to random changes and are closer to 0. **h**) The accuracy of predictions caps off after ~1000 features in the ground state. The x-axis shows how many uniformly randomly features were sampled for the ground state that was used to build and validate an Elastic net regression model the same as in D) (trained on n = 300, 3 samples per time point). The y-axis shows the R² as a measure of model accuracy. Of note, the Elastic net regression will shrink coefficients of features to 0 and thereby reduce the features relevant for the prediction further. (N = 10 independent repeats for Features Sizes<1000, N = 3 independent repeats otherwise; each with n = 300, 3 samples per time point). Boxplots are shown with the center line depicting the median, the box limits the bottom, respective top quartiles, and the whiskers the 1.5x interquartile range. **i**) The amount of stochastic variation sets the pace of aging. The Elastic net regression model was trained the same as in D) with stochastic variation sampled from *N*(µ = 0, *σ*^2^ = 0.05^2^) (n = 300, 3 samples per time point). Color-coded are different independent validation samples, generated from the same ground state, but with stochastic variation from different normal distributions. Samples with stochastic variation from a distribution with a narrower standard deviation (*N*(µ = 0, *σ*^2^ = 0.025^2^)) accumulate less noise and are predicted to age slower, that is the slope of the prediction is lower. Samples with stochastic variation from a distribution with a wider standard deviation (*N*(µ = 0, *σ*^2^ = 0.1^2^), *N*(µ = 0, *σ*^2^ = 0.2^2^)) accumulate noise faster, have a steeper slope of prediction, and reach the maximum age faster. The x-axis shows the true simulated age, that is the number of times stochastic variation was added to the ground state. The y-axis shows the prediction of the Elastic net regression model of the independent validation data. All 4 simulated datasets consist of n = 300, 3 samples per time point.

To investigate whether a stochastic process is sufficient to build an age predictor from any dataset, we simulated random data with an age range between 0 and 100. We used 2,000 random data points (features) uniformly distributed between 0 and 1 as the ground state. The ground state is motivated by the proposed ground zero of organismal aging^36^. Features in prediction models can be any quantifiable data type normalized to values between 0 and 1. To test whether accumulating normal-distributed stochastic variation over time enables the building of an age predictor, we independently added such variation to all features in the ground state 1 to 100 times (Extended Data Fig. 1a and Methods). We simulated six sets of samples, applying stochastic variation from 1 to 100 times, reflecting a potential lifespan range. Note that the range from 1 to 100 was chosen arbitrarily. Using 3 sets of 100 samples we trained an elastic net regression that predicts the simulated age; that is, the number of times stochastic variation was added. To validate the model, we used the 300 independent validation samples, starting with the same ground state but adding independent stochastic variation from the same distribution (Extended Data Fig. 1b). Although the stochastic variation application makes the data noisier in each time-step and appears to be countable, no predictor can be built because the validation samples lack any trend in the data (Extended Data Fig. 1c; Pearson correlation: −0.05). Stochastic variation contains negative and positive values that are equally likely, thus on average canceling out the variation precluding a trend or prediction. When, however, we used the above approach but constrained the values between 0 and 1 after adding the stochastic variation, we observed an almost perfect prediction with a Pearson correlation for the independent validation data of 0.99 (*P* < 1 × 10^−16^, full statistics of all analyses can be found in the Source Data) (Extended Data Fig. 1d). Thus, the model found a pattern in the simulated data allowing the prediction of how often stochastic variation was added to the ground state (simulated age) even in independent validation data. Importantly, this will potentially work for any dataset, because our simulated starting point (ground state) consists of uniformly random data between 0 and 1, and the stochastic variation added at each time-step is randomly chosen from a normal distribution; that is, it does not require any regulation or program.

**Fig. 1:**
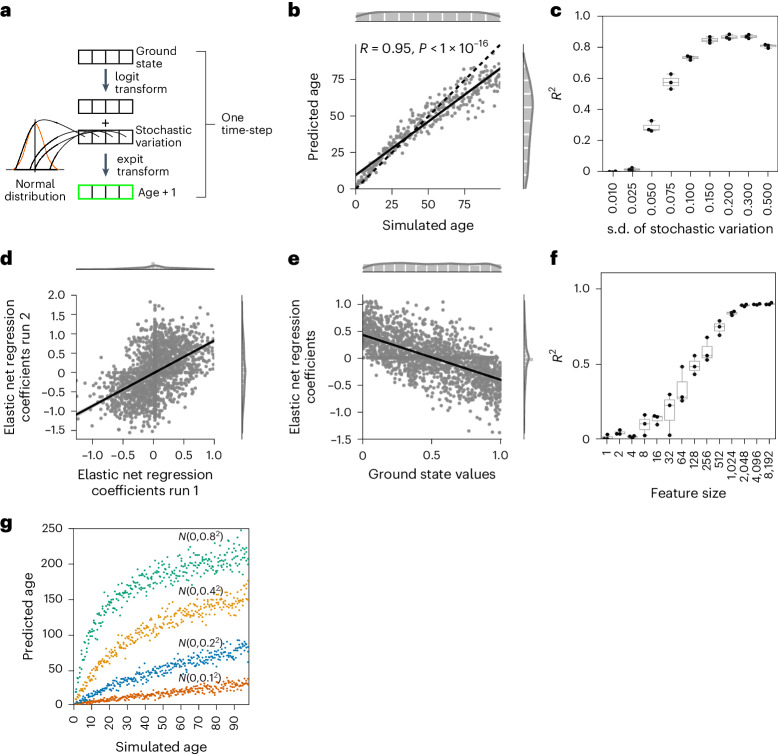
Normal-distributed stochastic variation accumulation simulations enable aging clock construction for simulated data. **a**, Sample generation explanation with logit transform. **b**, Accumulating stochastic variation in logit-transformed data enables accurate simulated age predictions. The *x* axis shows the number of times stochastic variation was added to the ground state and the *y* axis shows the prediction of the independent validation data (*n* = 300). **c**, Predictions of the independent validation data are robust to the stochastic variation distribution. The *x* axis shows the standard deviation of the normal distribution from which the stochastic variation was sampled and the *y* axis shows the *R*^2^ value of the independent validation data predictions (*N* = 3 independent repeats; each with *n* = 300 independent samples). **d**, Coefficients of independent models are highly correlated if trained on samples starting from the same ground. Shown are the coefficients of *N* = 2,000 features. **e**, The prediction in **b** is possible because of a regression to the mean. The *x* axis shows the starting values of the 2,000 features of the simulated ground state and the *y* axis shows the elastic net regression coefficients for the model in **b** (trained on *n* = 300). **f**, The accuracy of predictions plateaus after ~2,000 features in the ground state. The *x* axis shows how many features were randomly sampled for the ground state and the *y* axis shows *R*^2^ as a measure of model accuracy (*N* = 10 independent repeats for features sizes <1,000, *N* = 3 independent repeats otherwise; each with *n* = 300, 3 independent samples per time point). **g**, The amount of stochastic variation sets the pace of aging. The elastic net regression model was trained with stochastic variation sampled from *N*(*µ* = 0, *σ*^2^ = 0.2^2^) and tested on independent samples generated from the same ground state, but with varying degrees of stochastic variation (color-coded, as indicated in the panel). All simulated datasets consist of *n* = 300 independent samples. Boxplots in **c** and **f** are shown with the center line depicting the median, the box limits denote the bottom and top quartiles, and the whiskers indicate the 1.5× interquartile range.

To account for the non-normal distribution of values that are bounded by 0 and 1, we transformed the values before adding stochastic variation using the logit transform and transformed the data back via the expit (inverse-logit) transformation (Fig. 1a). A predictor built on these transformed data replicates the model in Extended Data Fig. 1d, further establishing the validity of accumulating stochastic variation in predicting age independent of whether a data transformation was used or not (Fig. 1b; Pearson correlation: 0.95).

The prediction accuracy of the independent validation data was robust to the distribution from which stochastic variation was sampled for the training and validation samples (Fig. 1c and Extended Data Fig. 1e). The logit-transformed data require a slightly higher data range from which the stochastic variation is sampled (Fig. 1c). Even predictions in which the age-related stochastic variation per time-step was smaller than the stochastic variation with which we varied the ground state for each sample (*N*(*µ* = 0, *σ*^2^ = 0.01^2^)), showed high accuracy; for example, the model trained on stochastic variation sampled from *N*(*µ* = 0, *σ*^2^ = 0.005^2^) per time-step still had a median *R*^2^ (coefficient of determination) value of 0.79 for prediction of the independent validation data (Extended Data Fig. 1e). This indicates that even a small amount of accumulating stochastic variation per time-step is enough for an accurate prediction.

During training, elastic net regression assigns a coefficient to each of the 2,000 features that can then be used to predict novel independent samples. The elastic net regression coefficients for the 2,000 features in our simulation in Fig. 1b and Extended Data Fig. 1d are reproducible in between independent runs with the same ground state (Fig. 1d and Extended Data Fig. 1f), indicating that even random stochastic variation patterns allow for robust predictions. Prediction is possible because of a regression to the mean, which is to be expected from a stochastic process with a data range limit (Fig. 1e and Extended Data Fig. 1g). Features starting close to 0 tend to increase after stochastic variation addition resulting in a positive elastic net coefficient, whereas features close to 1 tend to decrease resulting in a negative coefficient. Features starting around 0.5 in the ground state are more sensitive to noise because the added stochastic variation is equally likely to move in either direction leading, on average, to a cancelation of noise (Fig. 1e and Extended Data Fig. 1g).

The prediction accuracy of the amount of normal-distributed stochastic variation plateaus after ~2,000 features at an *R*^2^ value of around 0.97, showing that even models with a limited number of features are highly accurate (Fig. 1f and Extended Data Fig. 1h). Of note, elastic net regression shrinks the coefficients of some features to 0 and thereby further reduces the number of features. These results show that reproducible predictions are possible with fewer than 2,000 features (much fewer than are usually available in biological datasets involving any omics approaches), as long as there is accumulating stochastic variation and the data can be normalized between 0 and 1 (that is, predictions are not limited to DNA methylation or transcriptomic data).

We next wondered how a model trained on stochastic variation sampled from *N*(*µ* = 0, *σ*^2^ = 0.2^2^) would predict samples with different stochastic variation distributions. Choosing a standard deviation that is twice as large (*σ* = 0.4) also doubles the interval from which ~99.7% of stochastic variation values are sampled, which increases the amount of stochastic variation added in each time-step. Testing the model on data simulated with more stochastic variation per time-step resulted in a faster increase and plateau in the prediction, whereas a reduced stochastic variation level decreased the slope of the prediction (Fig. 1g and Extended Data Fig. 1i). Samples with more stochastic variation per time-step reach their maximum simulated age earlier. This analysis suggests that an increase in stochastic variation accelerates, whereas a decrease in stochastic variation decelerates the predicted aging process.

### Transcriptomic biological age prediction

**Fig. 2:**
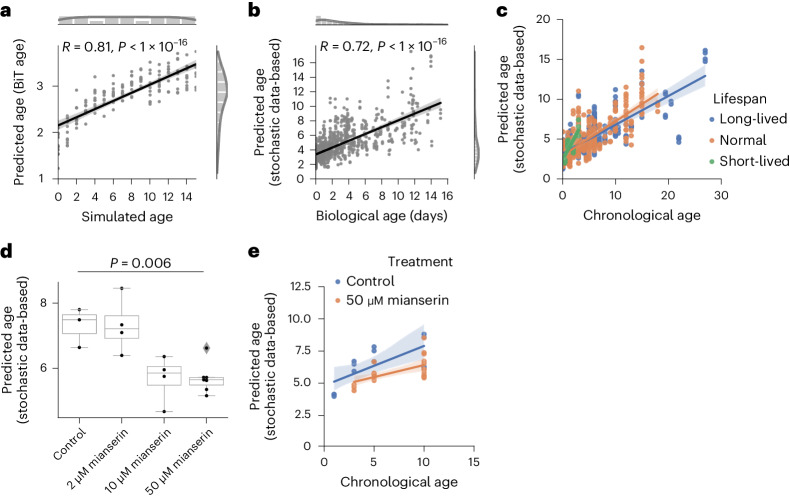
Normal-distributed stochastic variation accumulation simulations enable aging clock construction for transcriptomic data. **a**, Simulated age and BiT age^37^ predictions correlate significantly (Pearson correlation: 0.81, *P* = 5.99 × 10^−41^, two-sided test); *n* = 160, 10 independent samples per time point. Variation was sampled with a s.d. of 0.01. **b**, Predictions of a transcriptomic stochastic data-based clock correlate significantly (Pearson correlation: 0.72, *P* = 5.7 × 10^−150^, two-sided test) with the biological age of *n* = 993 independent RNA-seq from 61 independent public datasets (Supplementary Table 1). **c**, There is a significant association between the median lifespan and the predicted age of the clock used in **b** (median lifespan coefficient *P* = 0.015). Regression model fit with a 95% confidence interval (shadowed area) shown for long-lived (median lifespan >20 days, blue), short-lived (median lifespan <8 days, green) and normal-lived (orange) samples. **d**, Mianserin shows a dose-dependent decrease in the predicted age of the clock used in **b**. ANOVA (*P* = 0.006) with a two-sided Tukeyʼs post hoc test was used (50 μM mianserin versus control adjusted *P* = 0.026). Boxplots are as described in Fig. 1. **e**, Mianserin (50 μM) shows a lower predicted age over the whole time-course (two-way ANOVA treatment *P* = 7.3 × 10^−4^). Full statistics are available in the Source Data. The regression model fit with a 95% confidence interval (shadowed area) is shown for worms receiving 50 μM mianserin (orange) and control worms (blue).

**Extended Data Fig. 2:**
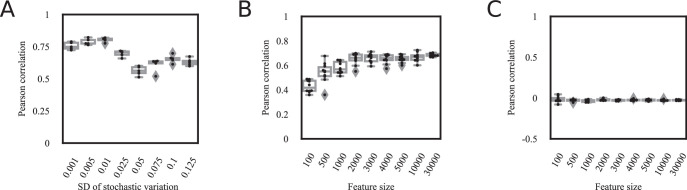
The effect of the feature size and the amount of stochastic variation on transcriptomic stochastic variation accumulation simulations. **a**) The BitAge predictions in Fig. 2a are robust to the distribution from which the stochastic variation is sampled. The x-axis shows the standard deviation of the normal distribution (centered at 0) from which stochastic variation for the simulations is sampled. The y-axis shows the Pearson correlation between the BitAge prediction of the simulated samples and the number of stochastic variation additions of the samples. Stochastic variation sampled from a normal distribution centered at 0 and a standard variation of 0.01 shows the highest Pearson correlation. N = 5 independent experiments are shown. Boxplots are shown with the center line depicting the median, the box limits the bottom, respective top quartiles, and the whiskers the 1.5x interquartile range. **b**) The feature size is largely irrelevant for the model in Fig. 2b). Predictions of Elastic net regression models trained on more than 100 features are significantly correlated with the biological age of *C. elegans* samples. The x-axis shows the number of randomly selected features, that is genes, for the ground state, which were subsequently used to generate data based on stochastic variations (see methods for details). These simulated samples were used to train the Elastic net regression. The y-axis shows the Pearson correlation between the biological age of the 993 independent samples (excluding the sample from which the ground state was sampled) and the prediction of the independent stochastic-data based model. N = 10 independent experiments are shown. Boxplots are shown with the center line depicting the median, the box limits the bottom, respective top quartiles, and the whiskers the 1.5x interquartile range. **c**) Verification of Extended Data Fig. 2b). Using the same approach as in Extended Data Fig. 2b, but with randomly shuffled biological ages of the *C. elegans* samples shows no significant correlation, indicating that biological age, and not a confounding variable is correlated with the predictions of the model based on simulated data. The x-axis shows the number of randomly selected features, that is genes, for the ground state, which were subsequently used to generate data based on stochastic variations (see methods for details. These simulated samples were used to train the Elastic net regression. The y-axis shows the Pearson correlation between the biological age of the 993 independent samples (excluding the sample from which the ground state was sampled) and the prediction of the stochastic-data based model. N = 10 independent experiments are shown. Boxplots are shown with the center line depicting the median, the box limits the bottom, respective top quartiles, and the whiskers the 1.5x interquartile range.

We next wondered whether an age predictor based on gene expression data applied to data with accumulation of stochastic variation would show a comparable correlation result. We have recently developed a highly accurate biological age predictor of *C. elegans* with the binarized transcriptome aging (BiT age) clock^37^. We defined the ground state as the biologically youngest adult RNA sequencing (RNA-seq) sample (GSM2916344)^38^ in our dataset and simulated stochastic variation similarly as explained in Extended Data Fig. 1a; that is, with (not empirically-estimated) normal-distributed variation. In accordance with our results in Fig. 1b and Extended Data Fig. 1d, BiT age predictions also correlate linearly with the amount of stochastic variation in the data (Fig. 2a; Pearson correlation: 0.81). The correlation is robust to the amount of stochastic variation added in each time-step with a peak in Pearson correlation of 0.81 at stochastic variation sampled from a normal distribution with a standard deviation of 0.01 (Extended Data Fig. 2a). This indicates that the predicted transcriptomic age of *C. elegans* correlates with age-dependent stochastic variation in the data.

Next, we wondered whether a stochastic data-based clock could predict the biological age of biological samples. Stochastic data-based clock predictions were significantly correlated (Pearson correlation: 0.72) with the biological age of 993 independent *C. elegans* RNA-seq samples from 61 independent public datasets for which the biological age could be calculated (Fig. 2b, Supplementary Table 1 and Methods). This prediction is robust to the number of features (genes) used in the simulation (Extended Data Fig. 2b). A permutation of the biological age does not correlate with the predicted simulated age (Extended Data Fig. 2c).

To test whether a stochastic age predictor could identify age acceleration and deceleration across a wide spectrum of aging interventions, we divided the 993 transcriptome samples into long-lived (median lifespan >20 days), normal-lived and short-lived (median lifespan <8 days). Plotting the predictions against the chronological age shows small but significant differences. A multivariate linear regression with the chronological age, the median lifespan and its interaction term shows a significant median lifespan effect with a negative coefficient; that is, a longer lifespan leads to a lower prediction based on the stochastic data-based clock (*P* = 0.015) (Fig. 2c). This indicates that accumulating stochastic variation scales mostly with chronological age, but also shows a significant lifespan effect (biological age prediction). A lifespan-extending treatment that was shown to reduce transcriptional drift (a measure of transcriptomic variance) is the anticonvulsant mianserin^39^. Consistent with reducing age-associated variation in gene expression, we found that mianserin dose-dependently decreases the predicted age with the stochastic data-based clock in independent data (Fig. 2d; one-way analysis of variance (ANOVA), *P* = 0.006; post hoc Tukey test 50 μM mianserin versus control, *P* = 0.03). Mianserin (50 μM) shows a (nonsignificant) lower slope as well as generally lower predicted values over a time-course (*P* = 7.3 × 10^−4^) compared with control samples (Fig. 2e). These results indicate that the stochastic transcriptomic data-based clock predictions of *C. elegans* can predict the chronological age and the biological age deceleration of a pharmacological intervention affecting transcription drift.

### Single-cell DNA methylation simulations

**Extended Data Fig. 3:**
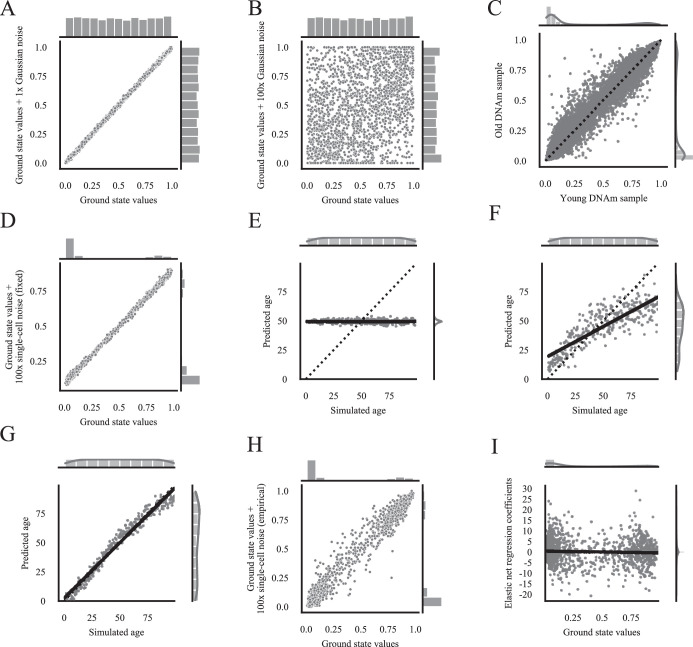
DNA methylation stochastic variation accumulation simulations. **a**) Comparison between the ground state on the x-axis, and the ground state (N = 2000 uniformly randomly sampled features between 0 and 1) after applying stochastic variation from *N*(µ = 0, *σ*^2^ = 0.05^2^), that is Gaussian noise, once on the y-axis. **b**) Comparison between the ground state on the x-axis, and the ground state (N = 2000 uniformly randomly sampled features between 0 and 1) after applying stochastic variation from *N*(µ = 0, *σ*^2^ = 0.05^2^), that is Gaussian noise, 100 times on the y-axis. **c**) Comparison of human blood DNA methylation data of the youngest (x-axis= GSM1007467) and oldest (y-axis= GSM1007832) subjects in the public dataset GSE41037 ref. ^78^. Every dot depicts a DNA methylation site (n = 21389). Values close to 0 and 1 show less variation than values closer to 0.5. **d**) Comparison of the ground state on the x-axis (2000 randomly sampled features from the youngest healthy sample (GSM1007467 ref. ^78^)) and the ground state after applying 100x single cell stochastic variation steps with a universal maintenance efficiency rate of 99.9 %, that is the maintenance efficiency rate is fixed to be the same for all features (y-axis). **e**) Starting single-cell simulations with a ground state consisting of 2000 features at 0.5 with a universal maintenance of 99 % allows no prediction. An Elastic net regression model was trained on n = 300 samples (3 samples per time point) starting from the same ground state in which all features were set to 0.5, and universal maintenance efficiencies ${E}_{m}$ and ${E}_{u}$ of 99 %. The x-axis shows the true simulated age, that is the number of times stochastic variation was added to the ground state. The y-axis shows the prediction of the Elastic net regression model of the independent validation data (n = 300, 3 samples per time point). The sides show the distribution of the samples. **f**) Starting single-cell simulations with a ground state consisting of 2000 features at 0.51 with a universal maintenance of 99 % allows for an accurate age prediction. The training and validation were done the same as in B) with the difference that all features in the ground state started at 0.51. (n = 300, 3 samples per time point). **g**) Starting single-cell simulations with a ground state consisting of 2000 features at 0.5 with biologically estimated maintenance rates allows for an accurate prediction. The training and validation were done the same as in B) with the difference that ${E}_{m}$ and ${E}_{u}$ values were estimated from biological data (see methods for details). (n = 300, 3 samples per time point). **h**) Comparison of the ground state on the x-axis (2000 randomly sampled features from the youngest healthy sample (GSM1007467 ref. ^78^)) and the ground state after applying 100x single cell stochastic variation steps (y-axis) with empirically estimated maintenance efficiency rates with the limits ${E}_{m}$ > 95 % and ${E}_{d}$ < 23 %. **i**) The prediction in Fig. 3f) is not due to a regression to the mean, different to Fig. 1. The x-axis shows the starting values of the 2000 randomly sampled features from the youngest healthy sample (GSM1007467 ref. ^78^) as the ground state, the y-axis the Elastic net regression coefficients for the model in Fig. 3f) (n = 300, 3 samples per time point). All ground state features can have positive as well as negative coefficients, indicating that the prediction is not based on a regression to the mean.

The most well-established aging clocks in mammals, including humans, are based on age-related changes in epigenetic CpG sites. We assessed whether simulations based on accumulating stochastic variation might be applicable to epigenetic data. Adding normally distributed stochastic variation once in the simulation in Fig. 1 did not change the simulated sample much from the ground state (Extended Data Fig. 3a), whereas adding stochastic variation 100 times led to a uniform distribution of features (Extended Data Fig. 3b). However, CpG methylation sites are typically under higher maintenance and are less noisy. Comparing biological DNA methylation data of young and old subjects shows that the methylation sites starting close to the extremes (0 or 1) indeed show less variance (Extended Data Fig. 3c).

**Fig. 3:**
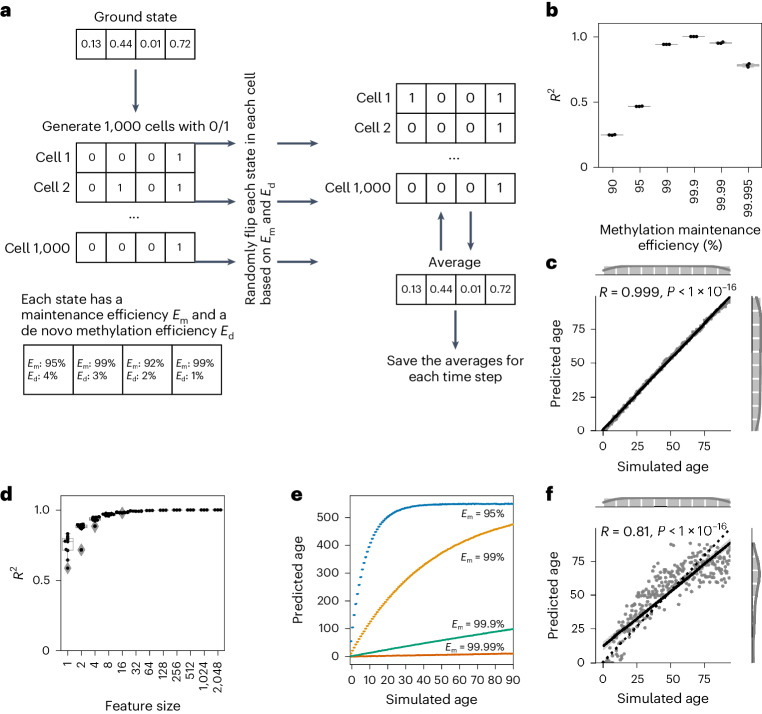
Single-cell DNA methylation stochastic variation accumulation simulations enable aging clock construction for simulated data. **a**, Explanation of single-cell simulations. **b**, The accuracy of the model is dependent on the methylation maintenance efficiency rate. A stochastic data-based clock was trained with 500 features and universal maintenance efficiencies *E*_m_ and *E*_d_, and was used to predict the simulated age of 300 independent validation samples. *N* = 3 independent experiments with different ground states are shown for each maintenance efficiency. **c**, Single-cell simulation of DNA methylation sites with *E*_m_ and *E*_u_ values of 99.9% allows us to build a clock with highly accurate predictions (*R*^2^ = 0.999) of independent validation data (*n* = 300). **d**, The accuracy of predictions with a universal maintenance efficiency rate of 99.9% plateaus after ~32 features with an *R*^2^ value of 0.99. *N* = 10 independent repeats for features sizes <1,000, *N* = 3 independent repeats otherwise; each with *n* = 300, 3 samples per time point. Boxplots in **b** and **d** are as described in Fig. 1. **e**, The maintenance efficiency rate sets the pace of aging. The stochastic data-based clock was trained with a maintenance efficiency of *E*_m_ = *E*_u_ = 99.9%, and tested on independent samples generated from the same ground state, but with varying maintenance efficiencies (color-coded, as indicated in the panel). All simulated datasets consist of *n* = 300 independent samples. **f**, Biologically estimated maintenance rates allow for highly accurate predictions. Site-specific *E*_m_ and *E*_u_ values were estimated from the data (Methods). The simulations were the same as in **c** but with site-specific maintenance rates (*n* = 300).

Instead of bulk data between 0 and 1, we next simulated ‘single-cell’ data for which each feature is binary, either methylated (1) or unmethylated (0) (Fig. 3a). Note that this is a simplification for diploid organisms; however, this should not affect the results because in theory the different alleles could be represented as different features in the simulations. It has been shown that a bulk methylation pattern at single CpG sites can be modeled using differential equations containing a methylation maintenance efficiency (*E*_m_; the probability that a methylated site stays methylated) and a de novo methylation efficiency (*E*_d_; the probability that an unmethylated site gets methylated; 1 − *E*_d_ is the maintenance efficiency of the unmethylated state (*E*_u_))^24^. These maintenance efficiencies describe the rate by which a CpG site does not alter per time-step. We simulated single-cell DNA methylation changes in a stochastic system over time, as depicted in Fig. 3a, using a variety of maintenance efficiencies (site-specific efficiencies that are either estimated from data, randomly chosen or universal efficiencies that are fixed to one value for all CpG sites).

First, we tested how a universal maintenance efficiency rate (the same rate for all 500 features) would affect the accuracy of the model (Fig. 3b). A high maintenance (*E*_m_ = 99.9%, *E*_d_ = 0.01%; that is, *E*_u_ = 99.9%) yielded almost perfect simulated age predictions (*R*^2^ = 0.999) on the independent validation data (Fig. 3b,c). A simulated age of 100 shows minimal deviation from the ground state, demonstrating high accuracy with small effect sizes (Extended Data Fig. 3d). Even maintenance rates of up to 99.995% resulted in a prediction with an *R*^2^ value of 0.78 (Fig. 3b). The predictor is robust in the number of features allowing for highly accurate age predictions with small feature sizes, whose accuracy plateaus after around 32 features (Fig. 3d). Training the model on *E*_m_ = 99.9% and testing it on data simulated with lower and respectively higher values of *E*_m_, showed that less maintenance accelerates, whereas higher maintenance decelerates the aging clock (Fig. 3e). These results indicate that even a high maintenance rate yields accurate age predictions, and that an increase in maintenance decelerates, whereas a decrease in maintenance accelerates the predicted age.

A maintenance rate of 99.9% for methylated as well as unmethylated sites leads to a regression to the equilibrium (0.5). Starting the simulation at equilibrium and at *E*_m_ = 99.9% did not allow for a prediction of the simulated age, because no regression to the equilibrium state is possible (Extended Data Fig. 3e; Pearson correlation: 0.05). However, a slight deviation to 0.51 for all starting values in the ground state led to an accurate simulated age prediction via a regression to the equilibrium state (Extended Data Fig. 3f; Pearson correlation: 0.95).

Similar to the universal maintenance model (Fig. 3b–d), accurate simulated age predictions are possible if *E*_m_ and *E*_d_ are empirically estimated from data (Methods and Fig. 3f; Pearson correlation: 0.81). The predictions plateau earlier than in Fig. 3c because of lower maintenance rates, leading to a quicker convergence to the site-specific equilibria (Extended Data Fig. 3e).

Site-specific *E*_m_ and *E*_d_ values allow accurate simulated age prediction even when starting at 0.5 (Extended Data Fig. 3g; Pearson correlation: 0.99). Such a site-specific regression away from the mean is still in line with stochasticity and entropic alterations. Although the site-specific maintenance rates give a framework in which each feature will change, the change itself is purely stochastic. Stochastic variation after 100 time-steps shows less variation in features starting close to 0 or 1 than in features starting close to 0.5 (Extended Data Fig. 3h), resembling the comparison of young and old human DNA methylation datasets (Extended Data Fig. 3c). Without site-specific stochastic variation predictions were driven by the regression to the mean (Fig. 1e and Extended Data Fig. 1g), whereas site-specific stochastic variation showed no correlation (Extended Data Fig. 3i), suggesting a regression away from the mean could be explained via a stochastic process, arguing against a recent report that suggested clock sites starting around 0.5 could not be entropic^32^.

In conclusion, accurate age predictors can be built by simulating DNA methylation changes purely with stochastic variation based on the maintenance efficiency rates of methylated and unmethylated sites. In addition, DNA methylation sites can have equilibria unequal to 0.5, allowing for a stochastic regression away from the mean, and even sites close to the site-specific equilibria can confer information for the aging clock.

### Public aging clocks

**Fig. 4:**
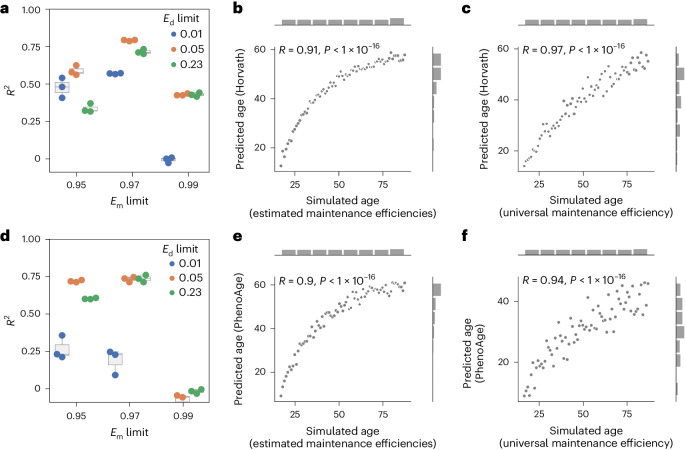
Epigenetic aging clock predictions correlate significantly with the amount of stochastic variation. **a**, The methylation maintenance efficiency limits affect the simulation and subsequent prediction with Horvath’s epigenetic clock^26^. The *R*^2^ value was calculated between the predicted epigenetic age by Horvath’s epigenetic clock^26^ and the simulated age. *N* = 3 independent repeats, each consisting of *n* = 73 independent samples. **b**, Horvath’s epigenetic age prediction^26^ of samples simulated based on biologically estimated maintenance rates with the limits *E*_m_ > 97% and *E*_d_ < 5%, correlates significantly with the simulated age. *N* = 73 independent samples. **c**, Horvath’s epigenetic age prediction^26^ of samples simulated based on a universal maintenance efficiency rate of 99% for all features, correlates significantly with the simulated age. *N* = 73 independent samples. **d**, Methylation maintenance efficiency limits affect the simulation and subsequent prediction with PhenoAge^40^. The *R*^2^ value was calculated between the predicted epigenetic age by PhenoAge^40^ and the simulated age. *N* = 3 independent repeats, each consisting of *n* = 73 independent samples. Boxplots in **a** and **d** are as described in Fig. 1. **e**, Biological age prediction with PhenoAge^40^ of samples simulated based on biologically estimated maintenance rates with the limits *E*_m_ > 97% and *E*_d_ < 5%, correlates significantly with the simulated age. *N* = 73 independent samples. **f**, Biological age prediction with PhenoAge^40^ of samples simulated based on a universal maintenance rate of 99% for all features, correlates significantly with the simulated age. *N* = 73 independent samples.

**Extended Data Fig. 4:**
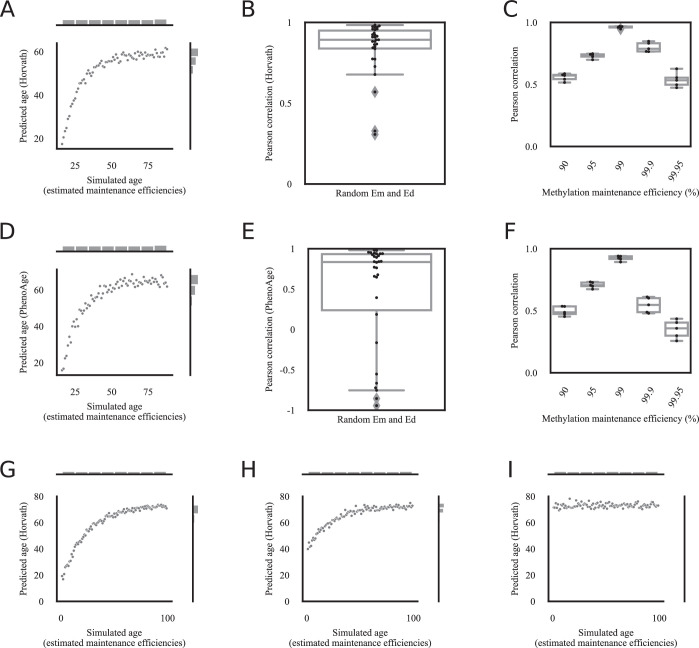
Epigenetic aging clock predictions correlate robustly with the amount of stochastic variation. **a**) Horvath’s epigenetic age prediction^26^ of samples simulated based on biologically estimated maintenance rates with the limits ${E}_{m}$ > 95 % and ${E}_{d}$ < 23 % starting from biological data from a young human blood sample (GSM1007467)^78^, correlates significantly with the simulated age, that is how often stochastic variation was applied to the ground state. N = 73 independent samples, one per age step from 16 to 88 are shown. **b**) Horvath’s epigenetic age prediction^26^ of samples simulated based on random maintenance rates within the limits ${97 \% < E}_{m}\le 100 \%$ and $0 \% \le {E}_{d} < 5 \%$ starting from biological data from a young human blood sample (GSM1007467)^78^, correlates significantly with the simulated age, that is how often stochastic variation was applied to the ground state. The y-axis shows the Pearson correlation between the simulated age and Horvath’s age prediction. N = 30 independent experiments with each n = 73 independent samples. Boxplots are shown with the center line depicting the median, the box limits the bottom, respective top quartiles, and the whiskers the 1.5x interquartile range. **c**) Pearson correlation of Horvath’s epigenetic age prediction^26^ of simulated data and the true simulated age for different universal methylation maintenance efficiencies. 5 independent experiments (each containing n = 73 independent samples, one per age step from 16 to 88) with different ground states are shown for each maintenance efficiency. Boxplots are shown with the center line depicting the median, the box limits the bottom, respective top quartiles, and the whiskers the 1.5x interquartile range. **d**) Biological age prediction with PhenoAge^40^ of samples simulated based on biologically estimated maintenance rates with the limits ${E}_{m}$ > 95 % and ${E}_{d}$ < 23 % starting from biological data from a young human blood sample (GSM1007467)^78^, correlates significantly with the simulated age, that is how often stochastic variation was applied to the ground state. N = 73 independent samples, one per age step from 16 to 88 are shown. **e**) Biological age prediction with PhenoAge^40^ of samples simulated based on random maintenance rates within the limits ${97 \% < E}_{m}\le 100 \%$ and $0 \% \le {E}_{d} < 5 \%$ starting from biological data from a young human blood sample (GSM1007467)^78^, correlates significantly with the simulated age, that is how often stochastic variation was applied to the ground state. The y-axis shows the Pearson correlation between the simulated age and PhenoAge’s age prediction. N = 30 independent experiments with each n = 73 independent samples. The boxplot is shown with the center line depicting the median, the box limits the bottom, respective top quartiles, and the whiskers the 1.5x interquartile range. **f**) Pearson correlation of biological age predictions with PhenoAge^40^ of simulated data and the true simulated age for different universal methylation maintenance efficiencies. 5 independent experiments (each containing n = 73 independent samples, one per age step from 16 to 88) with different ground states are shown for each maintenance efficiency. Boxplots are shown with the center line depicting the median, the box limits the bottom, respective top quartiles, and the whiskers the 1.5x interquartile range. **g**) Horvath’s epigenetic age prediction^26^ of samples simulated based on biologically estimated maintenance rates with the limits ${E}_{m}$ > 97 % and ${E}_{d}$ < 5 % starting from biological data from a young human blood sample age 16 (GSM1007467)^78^, correlates significantly with the simulated age, that is how often stochastic variation was applied to the ground state. The simulation is the same as in Extended Data Fig. 4a, but with a simulated age range from 0–99 for an easier comparison with Extended Data Fig. 4h, i. N = 100 independent samples, one per age step from 0 to 99 are shown. **h**) Horvath’s epigenetic age prediction^26^ of samples simulated based on biologically estimated maintenance rates with the limits ${E}_{m}$ > 97 % and ${E}_{d}$ < 5 % starting from biological data from a middle-aged human blood sample age 37 (GSM1007384)^78^, still correlates significantly with the simulated age, that is how often stochastic variation was applied to the ground state. The predicted age starts at a later time-point than the predictions in Extended Data Fig. 4g, and reaches the cap-off earlier. N = 100 independent samples, one per age step from 0 to 99 are shown. **i**) Horvath’s epigenetic age prediction^26^ of samples simulated based on biologically estimated maintenance rates with the limits ${E}_{m}$ > 97 % and ${E}_{d}$ < 5 % starting from biological data from an old human blood sample age 81 (GSM1007791)^78^, does not correlate significantly with the simulated age, that is how often stochastic variation was applied to the ground state. Starting the ground state at an old age does not allow for a correlation between the predicted epigenetic age and the amount of stochastic variation in the data, since the prediction already starts in the cap-off. N = 100 independent samples, one per age step from 0 to 99 are shown.

Next, we wondered whether published DNA methylation aging clocks might also mainly measure stochastic variation. Horvath’s pan-tissue DNA methylation clock^26^ predicts a linear increase in the amount of stochastic variation generated based on empirically estimated *E*_m_ and *E*_d_ values until it plateaus at a predicted age of around ~60 years (Extended Data Fig. 4a; Pearson correlation: 0.91). The time-steps in our simulations are arbitrary and not directly comparable with the predicted age, because our simulated age tracks how often we added stochastic variation, and the predicted age is the epigenetic age in years. We wondered whether we could estimate the range limits of the site-specific *E*_m_ and *E*_d_ such that the epigenetic age prediction of our simulated data would be as accurate as possible regarding the simulated age. We tested multiple combinations of limits for *E*_m_ and *E*_d_ and calculated *R*^2^ as a measure of accuracy between the predicted and simulated ages (Fig. 4a). Horvath’s epigenetic clock has the highest accuracy in predicting the simulated age with the limits 97% < *E*_m_ ≤ 100% and 0% ≤ *E*_d_ < 5%, suggesting higher site-specific maintenance with a narrower range for *E*_m_ and *E*_d_ than previously assumed (Fig. 4a). Indeed, the prediction with Horvath’s epigenetic clock plateaus later with these new limits (Fig. 4b; Pearson correlation: 0.91, compare Extended Data Fig. 4a). These results suggest that the site-specific maintenance rates are sufficient to explain the predictability of Horvath’s aging clock.

Randomly choosing *E*_m_ and *E*_d_ within the limits 97% < *E*_m_ ≤ 100% and 0% ≤ *E*_d_ < 5% allowed simulations with highly significant Pearson correlations also (median Pearson correlation: 0.89; Extended Data Fig. 4b). The same is even true if, instead of site-specific maintenance rates, all CpG sites were simulated with a universal maintenance efficiency of 99% that was not inferred from a biological sample and could therefore not be confounded (Fig. 4c; Pearson correlation: 0.97). The Pearson correlations are robust to the universal methylation maintenance efficiency, but peak at 99% (Extended Data Fig. 4c). A low maintenance efficiency of 90% reduces the Pearson correlation (Extended Data Fig. 4c) because the features reach equilibrium faster and therefore plateau more quickly (compare with Fig. 3b). A high maintenance efficiency of 99.95% reduces the Pearson correlation because of the reduced speed of convergence (Extended Data Fig. 4c). Notably, Horvath’s clock predicts an old age of 69.4 years for a dataset with DNA methylation levels of 0.5 for all CpG sites. These results suggest that no biologically inferred maintenance rate is required but instead indicates that stochastic variation is sufficient for age prediction.

Next, we tested the second-generation aging clock PhenoAge^40^ (Fig. 4d–f and Extended Data Fig. 4d–f). The previously assumed limits for *E*_m_ and *E*_d_ led to a similar linear increase, and early plateauing of the predicted PhenoAge (Extended Data Fig. 4d; Pearson correlation: 0.89). Improved limits (Fig. 4d,e), coincide with those estimated for Horvath’s clock. PhenoAge significantly correlates with the simulated age of samples simulated with random *E*_m_ and *E*_d_ within the limits (Extended Data Fig. 4e; median Pearson correlation: 0.84), or a universal maintenance efficiency of 99% (Fig. 4f; Pearson correlation: 0.94), which also was robust to the maintenance efficiency chosen (Extended Data Fig. 4f).

We next tested how ground states defined at different ages might affect the age simulations. Starting the ground state with a sample from a 16-year-old and simulating the addition of up to 100 stochastic variations results in a linear increase in predicted age (Extended Data Fig. 4g; Pearson correlation: 0.89). Starting from a 37-year-old, begins the prediction higher, shows a smaller linear increase in the predicted age and leads to a quicker arrival and longer time at the plateau (Extended Data Fig. 4h). Starting from an 81-year-old does not show a difference in the prediction upon stochastic variation, indicating that the ground state already contains as much stochastic variation as we would expect at the plateau (Extended Data Fig. 4i; Pearson correlation: 0.09). These results affirm that our simulations are robust to the choice of the ground state and that the predictions are scaled accordingly.

**Extended Data Fig. 5:**
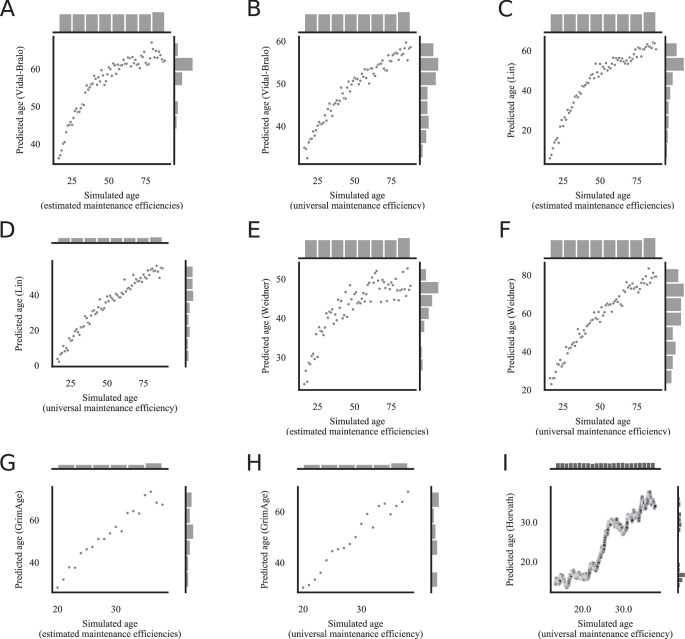
All tested epigenetic clock predictions correlate significantly with the amount of stochastic variation. **a**) Vidal-Bralo’s epigenetic age prediction^41^ of samples simulated based on biologically estimated maintenance rates with the limits ${E}_{m}$ > 97 % and ${E}_{d}$ < 5 % starting from biological data from a young human blood sample (GSM1007467)^78^, correlates significantly with the simulated age, that is how often stochastic variation was applied to the ground state. N = 73 independent samples, one per age step from 16 to 88 are shown. **b**) Vidal-Bralo’s epigenetic age prediction^41^ of samples simulated based on a universal maintenance rate of 99 % for all features (CpG sites) starting from biological data from a young human blood sample (GSM1007467)^78^, correlates significantly with the simulated age, that is how often stochastic variation was applied to the ground state. N = 73 independent samples, one per age step from 16 to 88 are shown. **c**) Lin’s epigenetic age prediction^42^ of samples simulated based on biologically estimated maintenance rates with the limits ${E}_{m}$ > 97 % and ${E}_{d}$ < 5 % starting from biological data from a young human blood sample (GSM1007467)^78^, correlates significantly with the simulated age, that is how often stochastic variation was applied to the ground state. N = 73 independent samples, one per age step from 16 to 88 are shown. **d**) Lin’s epigenetic age prediction^42^ of samples simulated based on a universal maintenance rate of 99 % for all sites starting from biological data from a young human blood sample (GSM1007467)^78^, correlates significantly with the simulated age, that is how often stochastic variation was applied to the ground state. N = 73 independent samples, one per age step from 16 to 88 are shown. **e**) Weidner’s epigenetic age prediction^43^ of samples simulated based on biologically estimated maintenance rates with the limits ${E}_{m}$ > 97 % and ${E}_{d}$ < 5 % starting from biological data from a young human blood sample (GSM1007467)^78^, correlates significantly with the simulated age, that is how often stochastic variation was applied to the ground state. N = 73 independent samples, one per age step from 16 to 88 are shown. **f**) Weidner’s epigenetic age prediction^43^ of samples simulated based on a universal maintenance rate of 99 % for all sites starting from biological data from a young human blood sample (GSM1007467)^78^, correlates significantly with the simulated age, that is how often stochastic variation was applied to the ground state. N = 73 independent samples, one per age step from 16 to 88 are shown. **g**) GrimAge’s epigenetic age prediction^44^ of samples simulated based on biologically estimated maintenance rates with the limits ${E}_{m}$ > 97 % and ${E}_{d}$ < 5 % starting from biological data from a young human blood sample generated with the 450k Human Methylation Beadchip (GSM990528)^80^, correlates significantly with the simulated age, that is how often stochastic variation was applied to the ground state. N = 20 independent samples are shown. **h**) GrimAge’s epigenetic age prediction^44^ of samples simulated based on a universal maintenance rate of 99 % for all sites starting from biological data from a young human blood sample generated with the 450k Human Methylation Beadchip (GSM990528)^80^, correlates significantly with the simulated age, that is how often stochastic variation was applied to the ground state. N = 20 independent samples are shown. **i**) Horvath’s epigenetic age prediction^26^ of samples simulated with Gillespies’s algorithm with a universal maintenance efficiency rate of 90 % for all features (CpG sites) starting from biological data from a young human blood sample (GSM1007467)^78^, correlates significantly with the simulated age, that is how often stochastic variation was applied to the ground state. Since the ground state was starting from a sample of a 16-year-old human, we set the starting point of the simulated age to 16. The time-steps in Gillespie’s algorithm are not fixed, in total N = 15999 simulations were computed.

All tested first-generation aging clocks^41–43^ and the second-generation aging clock GrimAge^44^ were significantly correlated with the simulated age irrespective of whether empirically estimated, random or universal maintenance rates were assumed (Extended Data Fig. 5a–h).

Using the Gillespie algorithm^45^ for event-based simulations, in which time-steps are not uniform but the time until the next event is calculated, recapitulates our results (Extended Data Fig. 5i; Pearson correlation: 0.98), indicating that our simulations are robust to the method used.

### Stochastic data-based aging clock

**Extended Data Fig. 6:**
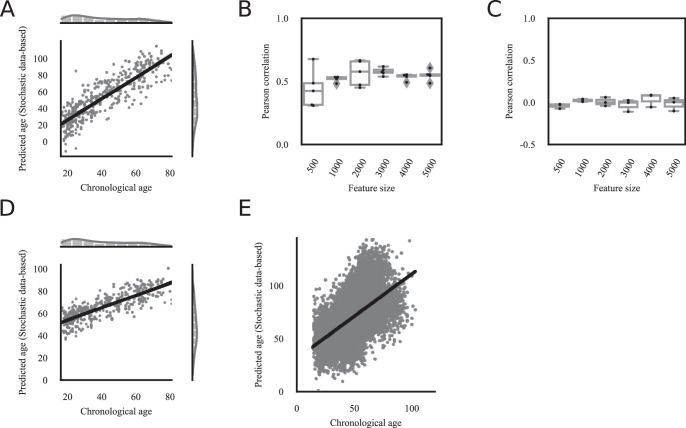
Human stochastic data-based clock predictions correlate significantly with the chronological age. **a**) The predictions of an Elastic net regression model based on simulated data, correlates significantly (Pearson correlation 0.87, p-value < 1e-16, two-sided test) with the chronological age of the independent healthy biological validation samples (GSE41037, n = 392)^78^. The simulated data is based on biologically estimated maintenance rates starting with Horvath’s epigenetic clock CpG sites from biological data from a young human blood sample. The x-axis shows the chronological age of the subjects from which blood DNA methylation data was processed. The y-axis shows the predicted simulated age, that is the prediction how often stochastic variation was added to the ground state and is therefore on a different scale and unit than the x-axis. **b**) The feature size is largely irrelevant for stochastic data-based models in Extended Data Fig. 6a. Predictions of Elastic net regression models trained on more than 500 random CpG sites (features) are significantly correlated with the chronological age. The x-axis shows the number of randomly selected features, that is CpG sites, for the ground state, which were subsequently used to generate data based on stochastic variations (see methods for details). These simulated samples were used to train the Elastic net regression. The y-axis shows the Pearson correlation between the chronological age of the n = 392 healthy samples in GSE41037 ref. ^78^ (excluding the sample from which the ground state was sampled, and the oldest sample from which maintenance efficiencies were estimated) and the prediction of the independent stochastic-data based model. N = 5 independent experiments are shown. Boxplots are shown with the center line depicting the median, the box limits the bottom, respective top quartiles, and the whiskers the 1.5x interquartile range. **c**) Verification of Extended Data Fig. 6b). Using the same approach as in Extended Data Fig. 6a, but with randomly shuffled chronological ages shows no significant correlation, indicating that chronological age, and not a confounding variable is correlated with the predictions of the model based on simulated data. The x-axis shows the number of randomly selected features, that is CpG sites, for the ground state, which were subsequently used to generate data based on stochastic variations (see methods for details). These simulated samples were used to train the Elastic net regression. The y-axis shows the Pearson correlation between the permuted chronological age of healthy samples in GSE41037 ref. ^78^ (excluding the sample from which the ground state was sampled, and the oldest sample from which maintenance efficiencies were estimated) and the prediction of the stochastic-data based model. N = 3 independent experiments are shown. Boxplots are shown with the center line depicting the median, the box limits the bottom, respective top quartiles, and the whiskers the 1.5x interquartile range. **d**) The same analysis as in Fig. 5a, but the simulated stochastic data were additionally cell-type corrected and then used to train the clock (Pearson correlation 0.81, p < 1e-16, two-sided test). **e**) The validation of the stochastic data-based clock in Fig. 5 A on 11,146 independent samples from 15 independent datasets (GSE84727, GSE87571, GSE80417, GSE40279, GSE87648, GSE42861, GSE50660, GSE106648, GSE179325, GSE210254, GSE210255, GSE72680, GSE147740, GSE55763, GSE117860) shows a highly significant correlation (Pearson correlation 0.57, p-value < 1e-16).

We next aimed to address whether a clock built on simulated DNA methylation data (Methods) could predict the chronological age of mammalian biological samples. A simulated training dataset with the CpG sites from Horvath’s epigenetic clock led to a significant Pearson correlation of 0.87 (*P* < 1 × 10^−16^) for chronological age and the predicted simulated age (Extended Data Fig. 6a). This linear correlation holds for randomly chosen CpG sites and is robust across different feature sizes (Extended Data Fig. 6b), whereas randomly permuting the chronological age of samples leads to nonsignificant correlations (Extended Data Fig. 6c).

**Fig. 5:**
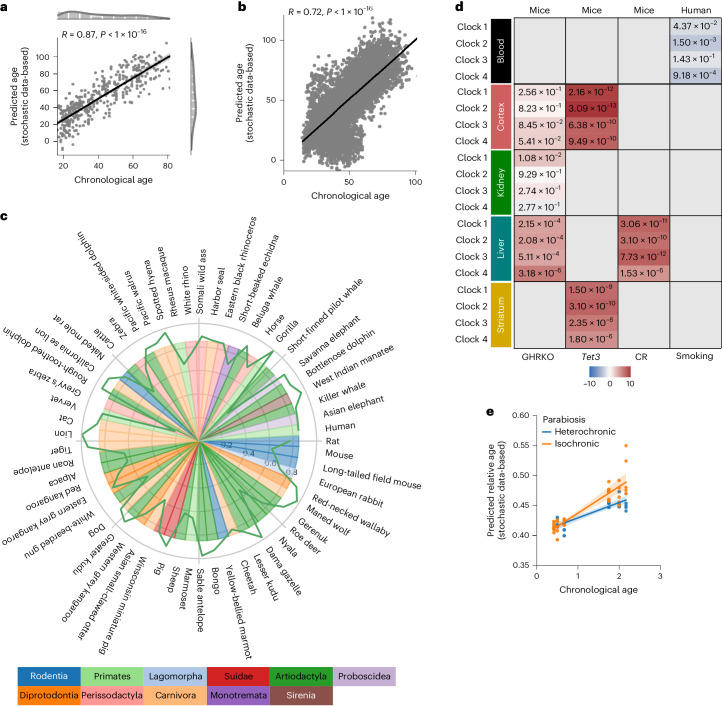
Single-cell DNA methylation stochastic variation accumulation simulations enable aging clock construction for pan-mammalian chronological and biological age predictions. **a**, The predictions of a stochastic data-based clock correlate significantly (Pearson correlation: 0.87, *P* < 1 × 10^−16^, two-sided test) with the chronological age of the cell-type corrected independent healthy biological validation samples (GSE41037, *n* = 392)^78^. **b**, Validation of the stochastic data-based clock starting from a fetal sample (GSM4682890) on 11,146 independent samples from 15 independent datasets (GSE84727, GSE87571, GSE80417, GSE40279, GSE87648, GSE42861, GSE50660, GSE106648, GSE179325, GSE210254, GSE210255, GSE72680, GSE147740, GSE55763, GSE117860) shows a significant correlation (Pearson correlation: 0.72, *P* < 1 × 10^−16^, two-sided test). **c**, Circle plot showing the Pearson correlation between the relative age of blood samples of the corresponding species and the predictions of clock 1 as a green line around the circle. Species are shown for which at least five blood samples were available in the dataset GSE223748. The colors within the circle show the taxonomic order of the corresponding species, as listed on the left-hand side. **d**, Validation of Clocks 1–4 on interventions with known lifespan effects in mouse and humans. Age-matched GHRKO mice with 30 normal (12 liver, 12 kidney, 6 cerebral cortex) and 29 GHRKO (11 liver, 12 kidney, 6 cerebral cortex) samples^15^; *Tet3*-knockout mice with 28 normal (14 striatum, 14 cerebral cortex) and 16 *Tet3* (8 striatum, 8 cerebral cortex) samples^15^; 36 CR mice with 59 normal mice^15^; and the effect of smoking on human aging^91^. The color gradient for mice is based on the sign of the *t*-test, the color of the human data is based on the interaction coefficient. The annotated values show the adjusted false discovery rate. **e**, Independent validation of clock 1 on parabiosis in young and old mice (GSE224361). Liver samples of mice that received either isochronic (orange) or heterochronic (blue) parabiosis are shown. A multivariate regression shows a significant age variable (*P* < 1 × 10^−16^) and interaction variable (*P* = 1.22 × 10^−3^). Full statistics are given in the Source Data. The regression model fit with a 95% confidence interval (shadowed area) is shown.

To exclude any potentially confounding effects of cell-type heterogeneity^46^, we estimated cell-type composition to subsequently correct the biological samples to obtain cell-type heterogeneity-adjusted CpG beta values. Using cell-type corrected data did not affect the performance of the stochastic data-based clock (Fig. 5a; Pearson correlation 0.87, *P* < 1 × 10^−16^), and an additional cell-type correction of the simulated samples still showed a Pearson correlation of 0.81 (*P* < 1 × 10^−16^) indicating highly correlated predictions of the biological samples (Extended Data Fig. 6d). In addition, we used a multivariate linear regression of the form:

$${\mathrm{{Age} \approx {PredictedAge}+{CellTypeFractions}}}.$$

This multivariate linear regression approach also showed a significant predicted age variable (*P* < 1 × 10^−16^, Source Data) for the predictions of the stochastic data-based clock. These results indicate that cell-type heterogeneity does not have a major role in the predictive power of stochastic variation accumulation.

We further probed for potential confounding effects by expanding the analysis to 11,146 independent whole blood or peripheral blood leukocyte samples from 15 different datasets. Stochastic data-based prediction of those samples still resulted in a Pearson correlation of 0.57 (*P* < 1 × 10^−16^) (Extended Data Fig. 6e).

**Extended Data Fig. 7:**
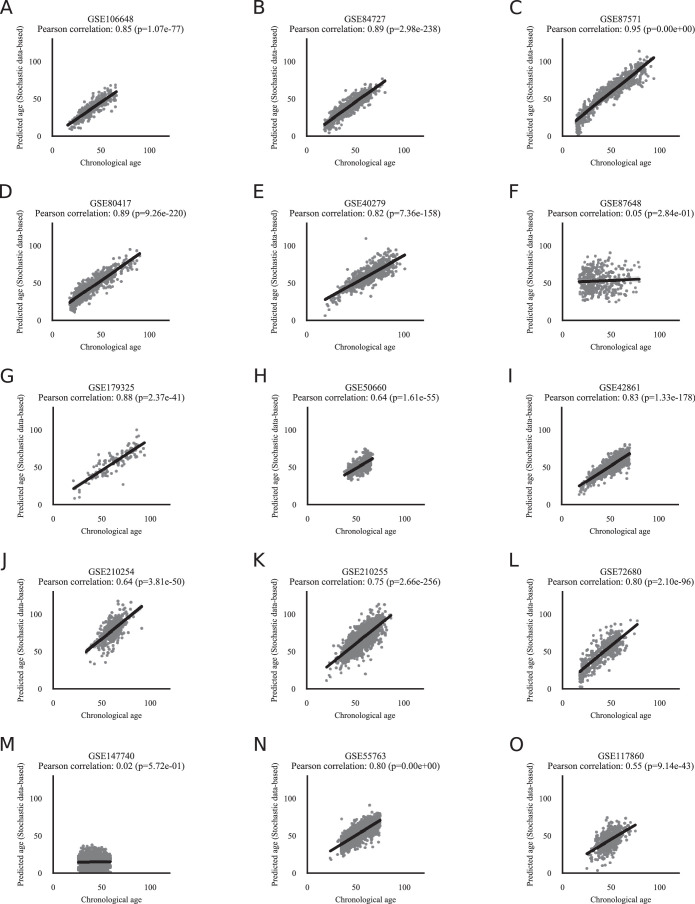
Human stochastic data-based clock predictions correlate significantly with the chronological age of independent validation data. The validation of the stochastic data-based clock starting from a fetal sample (GSM4682890) on 11,146 independent samples from 15 independent datasets **a**) GSE106648, **b**) GSE84727, **c**) GSE87571, **d**) GSE80417, **e**) GSE40279, **f**) GSE87648, **g**) GSE179325, **h**) GSE50660, **i**) GSE42861, **j**) GSE210254, **k**) GSE210255, **l**) GSE72680, **m**) GSE147740, **n**) GSE55763, **o**) GSE117860. See Fig. 5b for a combined plot. The Pearson correlation and its p-value, calculated with a two-sided test, are shown in the figure panels.

**Extended Data Fig. 8:**
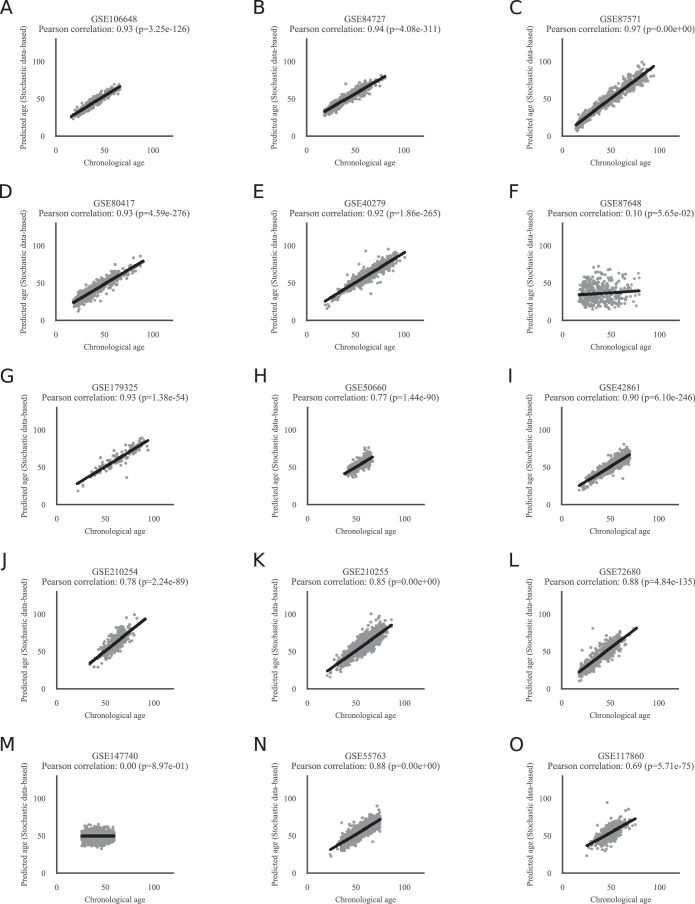
Horvath’s epigenetic age prediction results for the same 15 datasets. Horvath’s epigenetic age prediction on the same 11,146 samples from 15 independent datasets used in Extended Data Fig. 7. **a**) GSE106648, **b**) GSE84727, **c**) GSE87571, **d**) GSE80417, **e**) GSE40279, **f**) GSE87648, **g**) GSE179325, **h**) GSE50660, **i**) GSE42861, **j**) GSE210254, **k**) GSE210255, **l**) GSE72680, **m**) GSE147740, **n**) GSE55763, **o**) GSE117860. Note that GSE40279 and GSE42861 were used during test and training in Horvath’s original publication. Similar to Extended Data Fig. 7GSE87648 and GSE147740 do not show any correlation between the predicted and the chronological age. The Pearson correlation and its p-value, calculated with a two-sided test, are shown in the figure panels.

When instead of an adolescent ground state, we initiated the stochastic data-based clock with a fetal sample the Pearson correlation improved to 0.72 (Fig. 5b), with 9 of 15 datasets reaching correlations ≥0.8 (Extended Data Fig. 7). By comparison, Horvath’s original clock predicts the same samples with a Pearson correlation of 0.85, and 10 of 15 datasets with a correlation ≥0.8 (Extended Data Fig. 8).

In conclusion, our analysis shows that simulating epigenetic stochastic data starting from one young biological sample with site-specific maintenance rates, allows significantly correlated predictions with the chronological age of independent biological samples.

### Biological age prediction

Recently, a pan-mammalian clock suggested that instead of stochastic damage accumulation, aging might be a consequence of a developmental process because the clock sites were associated with genes implicated in developmental gene regulation^15^. To assess whether stochastic variation accumulation might also allow a prediction of the biological age, we next investigated the predictive power of a stochastic data-based clock on the data from the Mammalian Methylation Consortium^15,35,47^.

**Extended Data Fig. 9:**
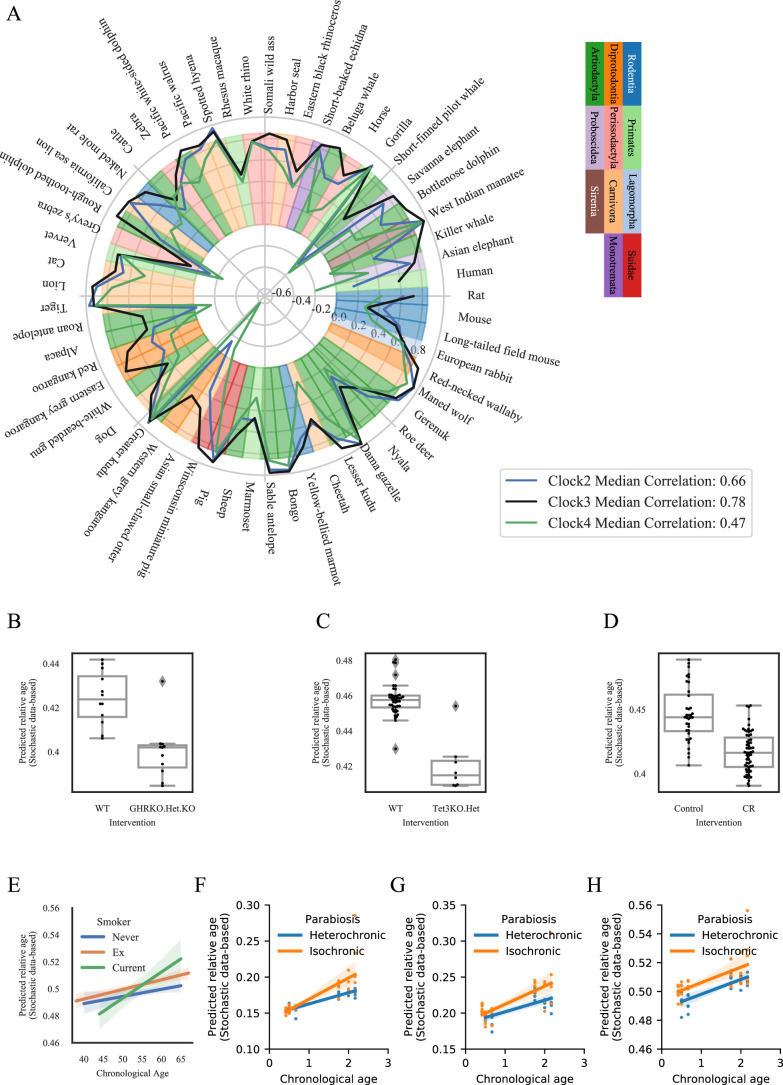
Stochastic data-based clock predictions correlate significantly with the chronological and biological age of pan-mammalian data. **a**) The same circle plot as in Fig. 5c, but for Clock 2–4. The Pearson correlation of the relative age of all blood samples of a given species and their predicted age of the stochastic data-based clocks are shown as lines around the circle. Species are shown for which at least 5 blood samples were available. The species are clock-wise sorted by maximum lifespan, starting with *Rattus norvegicus* (3.8 years) in the center right, and ending with *Homo sapiens* (122.5 years). The colors within the circle show the taxonomic order of the corresponding species, as listed on the right side. Clock 2 (99% maintenance rate for all CpG sites used in Lu’s pan-mammalian relative age clock^15^), Clock 3 (CpG site-specific empirically estimated maintenance rates from the oldest sample of *Tursiops truncatus* for all 37554 CpG sites), and Clock 4 (99% maintenance rate for all 37554 CpG sites) correlate on average highly significantly. **b**) Example comparison for Fig. 5d. Predictions of Clock 1 for GHRKO (n = 11 biologically independent samples) vs. WT (n = 12 biologically independent samples) liver samples show significantly lower values for GHRKO samples (two-sided adjusted p-value 2.15e-04, full statistics in **Source Data 1**). Boxplots are shown with the center line depicting the median, the box limits the bottom, respective top quartiles, and the whiskers the 1.5x interquartile range. **c**) Example comparison for Fig. 5d. Predictions of Clock 1 for *Tet3* (n = 8 biologically independent samples) vs. WT (n = 44 biologically independent samples) cerebral cortex samples show significantly lower values for *Tet3* samples (two-sided adjusted p-value 2.16e-12, full statistics in **Source Data 1**). Boxplots are shown with the center line depicting the median, the box limits the bottom, respective top quartiles, and the whiskers the 1.5x interquartile range. **d**) Example comparison for Fig. 5d. Predictions of Clock 1 for calorie restricted (CR) (n = 59 biologically independent samples) vs. normal fed (n = 36 biologically independent samples) liver samples show significantly lower values for CR samples (two-sided adjusted p-value 3.06e-11, full statistics in **Source Data 1**). Boxplots are shown with the center line depicting the median, the box limits the bottom, respective top quartiles, and the whiskers the 1.5x interquartile range. **e**) Example comparison for Fig. 5d. Current-smoker vs. ex-smoker vs. never-smoker aging trajectories are color-coded. The lines show the linear regression model fit of Seaborn’s lmplot function^88^, and the shadow around the lines the 95% confidence interval. Current-smoker show a steeper aging trajectory (slope) compared to never- or ex-smoker. **f**) The same as Fig. 5e, but for Clock 2. A multivariate regression of chronological age, the parabiosis treatment, and the interaction shows a significant age variable (p = 6.11e-12), and interaction variable (p = 1.13e-02). The regression model fit with a 95% confidence interval (shadowed area) is shown. **g**) The same as Fig. 5e, but for Clock 3. A multivariate regression of chronological age, the parabiosis treatment, and the interaction shows a significant age variable (p = 5.6e-09). The regression model fit with a 95% confidence interval (shadowed area) is shown. **h**) The same as Fig. 5e, but for Clock4. A multivariate regression of chronological age, the parabiosis treatment, and the interaction shows a significant age variable (p = 1.29e-06). The regression model fit with a 95% confidence interval (shadowed area) is shown. Full statistics can be found in **Source Data 1**.

We used four stochastic clocks starting from the youngest blood sample from *Tursiops truncatus* with different maintenance rates (Methods). All four clocks are on average highly significantly correlated with independent data, even from different species (Fig. 5c and Extended Data Fig. 9a), demonstrating that even one biological sample alone with simulated stochastic variation accumulation is sufficient to build aging clocks that are strongly correlated with the relative age of a variety of mammalian species.

Lu et al. further validated their clock on interventions that are known to slow biological age^15^. Applying our stochastic data-based clocks (clocks 1–4) on independent intervention data predicts significant age deceleration for growth hormone receptor knockout (GHRKO), mutant *Tet3* or calorie-restricted (CR) mice after multiple test correction (Fig. 5d and Extended Data Fig. 9b–d). Each intervention group showed, on average, strong effect sizes for all four clocks (see Source Data for full statistics). GHRKO liver samples have a Cohen’s *d* of 1.96 for clock 1 (Extended Data Fig. 9b), Tet3 mutant cerebral cortex samples have a Cohen’s *d* of 3.7 for clock 1 (Extended Data Fig. 9c) and CR liver samples have a Cohen’s *d* of 1.65 (Extended Data Fig. 9d). In a dataset of human smokers, previous smokers and never smokers our stochastic clocks predict a significant age acceleration trajectory in the smokers over the study course as calculated by a multivariate regression analysis (Fig. 5d and Extended Data Fig. 9e). We further validated our four clocks on an independent dataset on parabiosis in young and old mice^48^. A multivariate regression analysis showed that the predictions of clocks 1–4 are all highly significantly correlated with the chronological age (Fig. 5e (*P* = 7.8 × 10^−18^) and Extended Data Fig. 9f–h (*P* = 6.1 × 10^−12^, 5.6 × 10^−9^ and 1.3 × 10^−6^ respectively). Clocks 1 and 2 additionally showed a significant interaction term, indicating that heterochronic parabiosis in old mice leads to a younger predicted age compared with isochronic parabiosis, whereas there is no difference in young mice. These results further validate the chronological age prediction in independent datasets and corroborate that biological age is robustly predictable with accumulating stochastic variation.

**Fig. 6:**
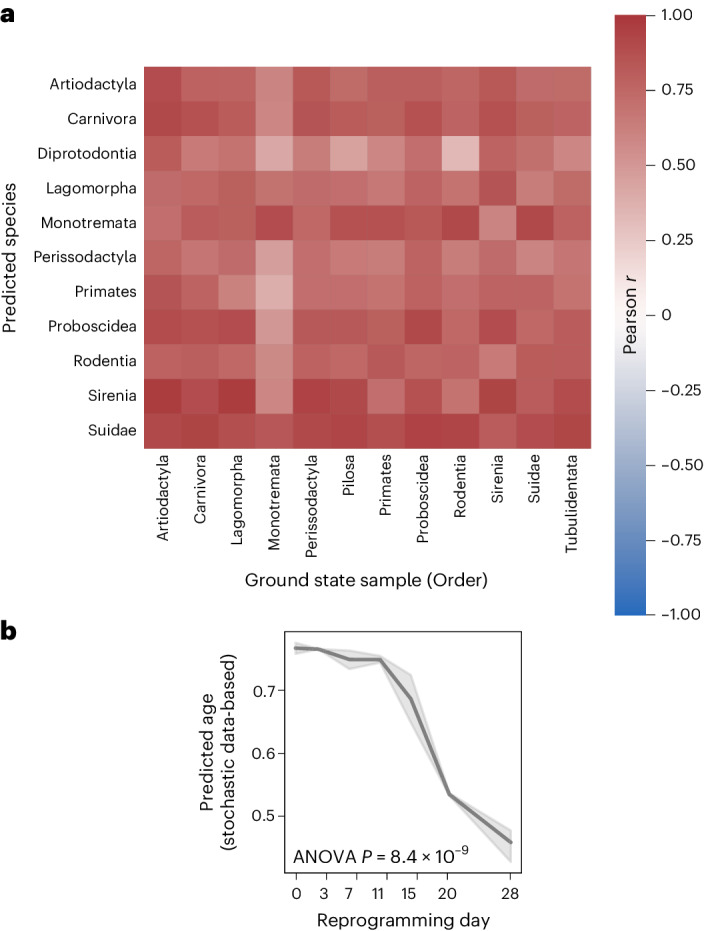
Single-cell DNA methylation stochastic variation accumulation simulations enable predictions for various species and reprogramming. **a**, Heatmap showing median Pearson correlations of species in the same taxonomic order between the predicted age of clock 1 trained on the youngest blood sample from species of the corresponding taxonomic order in the columns (Artiodactyla: *Tursiops truncatus*; Carnivora: *Odobenus rosmarus divergens*; Lagomorpha: *Oryctolagus cuniculus*; Monotremata: *Tachyglossus aculeatus*; Perissodactyla: *Equus caballus*; Pilosa: *Choloepus hoffmanni*; Proboscidea: *Loxodonta africana*; Rodentia: *Marmota flaviventris*; Sirenia: *Trichechus manatus*; Suidae: *Sus scrofa*; Tubulidentata: *Orycteropus afer*) and the relative age for all species in the rows. Values are shown for tissues and species for which at least five samples were available. **b**, The stochastic data-based clock in Fig. 5c was used on an independent reprogramming time-course dataset of human dermal fibroblasts (GSE54848)^49^. One-way ANOVA, *P* = 8.36 × 10^−9^ (statistics are shown in the Source Data). The line plot shows the mean values with a 95% confidence interval (shadowed area).

**Extended Data Fig. 10:**
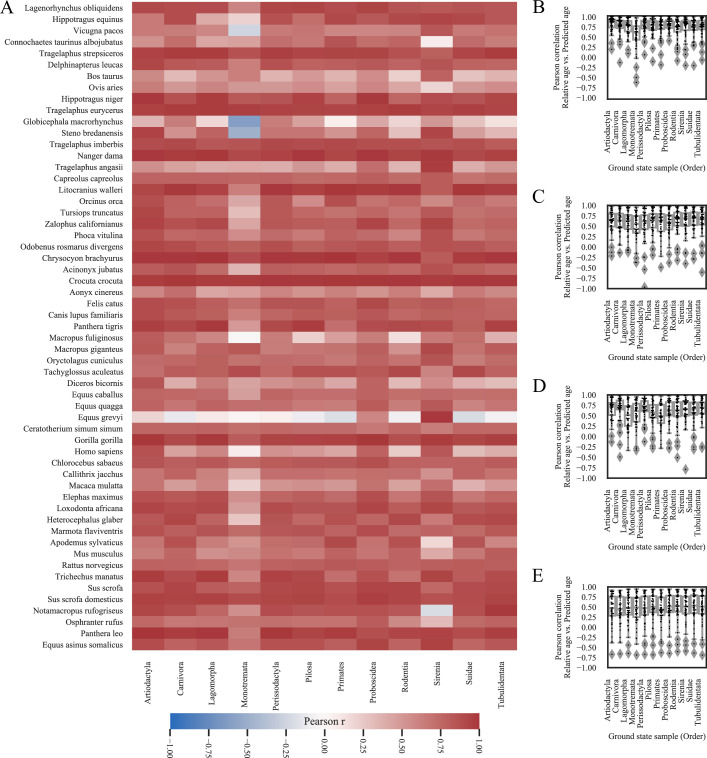
Stochastic data-based clock predictions for pan-mammalian data are robust to the choice of the ground state species. **a**) Heatmap showing Pearson correlations between the predicted age of Clock 1 trained on the youngest blood sample from species of the corresponding taxonomic order in the columns (Artiodactyla: *Tursiops truncatus*, Carnivora: *Odobenus rosmarus divergens*, Lagomorpha: *Oryctolagus cuniculus*, Monotremata: *Tachyglossus aculeatus*, Perissodactyla: *Equus caballus*, Pilosa: *Choloepus hoffmanni*, Proboscidea: *Loxodonta africana*, Rodentia: *Marmota flaviventris*, Sirenia: *Trichechus manatus*, Suidae: *Sus scrofa*, Tubulidentata: *Orycteropus afer*) and the relative age for all species in the rows. The Artiodactyla column corresponds to Fig. 5c. Values are shown for tissues and species for which at least 5 samples were available. **b**) The box-plots show the distribution of Pearson correlation values of Extended Data Fig. 10a. Clock 1 trained on samples starting from a Monotremata ground state with accumulating variation show on average a lower accuracy. For each of the 12 clocks (based on a different ground state as shown on the x-axis) the n = 57 biologically independent species orders (as indicated in Extended Data Fig. 10a) are shown as dots. Boxplots are shown with the center line depicting the median, the box limits the bottom, respective top quartiles, and the whiskers the 1.5x interquartile range. **c**) The same as Extended Data Fig. 10b but for Clock 2 trained with 99.99% maintenance rate for all sites of Lu’s pan-mammalian relative age-clock. For each of the 12 clocks (based on a different ground state as shown on the x-axis) the n = 57 biologically independent species orders (as indicated in Extended Data Fig. 10a) are shown as dots. Boxplots are shown with the center line depicting the median, the box limits the bottom, respective top quartiles, and the whiskers the 1.5x interquartile range. **d**) The same as Extended Data Fig. 10b but for Clock 3 trained on empirically estimated maintenance rates from the species specified in Extended Data Fig. 10a for all 37443 CpG sites. For each of the 12 clocks (based on a different ground state as shown on the x-axis) the n = 57 biologically independent species orders (as indicated in Extended Data Fig. 10a) are shown as dots. Boxplots are shown with the center line depicting the median, the box limits the bottom, respective top quartiles, and the whiskers the 1.5x interquartile range. **e**) The same as Extended Data Fig. 10b but for Clock 4 train with 99.99% maintenance rate for all 37443 CpG sites. For each of the 12 clocks (based on a different ground state as shown on the x-axis) the n = 57 biologically independent species orders (as indicated in Extended Data Fig. 10a) are shown as dots. Boxplots are shown with the center line depicting the median, the box limits the bottom, respective top quartiles, and the whiskers the 1.5x interquartile range.

To assess the effect of the ground state on predictions we built clocks for 12 different species orders, resulting on average in highly significantly correlations with values ranging from 0.6 for clock 1 starting from a Monotremata sample to 0.85 for clock 1 starting from a Artiodactyla sample (Fig. 6a and Extended Data Fig. 10a,b). Clocks 2–4 show similar results (Extended Data Fig. 10c–e). A clock built from the ground state of one order does not improve the prediction accuracy of species within the same order on average (Fig. 6a).

To assess whether ‘age-reversal’ could be measured by a stochastic data-based clock, we applied it to an independent reprogramming time-course of human dermal fibroblasts^49^. Despite differences in species, tissue-type and platform, a rejuvenation trajectory became evident, with a decreasing predicted age starting from 11 days of intermediate reprogramming and reaching the final lowest predicted age at 28 days (Fig. 6b; one-way ANOVA, *P* = 8.4 × 10^−9^). These results show that the stochastic data-based clock could identify study/tissue- and platform-independent signatures of age and captures biological aging as shown by the gradual decrease in the predicted age over the reprogramming time-course, as well as correctly predicted biological age differences in interventions.

## Discussion

During aging a range of biomolecular parameters show increased ‘noise’ such as stochastic DNA methylation drifts, degrading transcriptional networks in mouse muscle stem cells^50^ and increased cell-to-cell gene expression variation^51^. Transcriptomic variation can result from intrinsic (biochemical fluctuations and transcriptional bursting)^52^ and extrinsic noise such as stochastic DNA damage^53^. Predominantly affecting long genes^54^, transcription-blocking DNA lesions might explain the age-associated systemic transcript-length imbalance^55,56^. The role of stochasticity in transcription remains subject to debate as a recent study reported a lack of evidence for increased transcriptional single-cell noise in aged tissues^57^.

Stochastic changes occur during DNA methylation site copying or maintenance, like DNA repair and subsequent DNMT1 recruitment^58^, or in DNA replication^59^ because replication timing during S-phase itself has been shown to affect methylation maintenance levels^60^. The information-theoretic view of the epigenome^23^ suggests that higher maintenance, and therefore lower information loss, consumes more energy and is focused on more crucial regions of the genome.

The increased entropy with aging has been associated with higher hemi-methylation^23^, is correlated with chronological age, and longer-lived mice showed a lower entropy at age-related CpGs^61^, which are enriched in transcription factors and regulators of development and growth^62^. The EMS theory^26^ postulates that age-related epigenetic changes are the footprint of an imperfect maintenance system, leading to an increase in errors over time. CpG maintenance in genomic regions that are important for development might become less relevant during aging, leading to faster accumulation of stochastic variation. It was suggested that only 10% of CpG sites are driven by biological stochastic variation^63^. Our single-cell simulation results, by contrast, are in line with a recent report showing that a majority of CpG sites change stochastically^33^ even though only ~500 CpG sites could be analyzed because of the low coverage of single-cell data^64^.

The most trivial model of a stochastic process that can potentially be used for age prediction is a process that starts at a ground state of all 0s and has a certain low probability of switching to 1. Such a system will inevitably accrue changes (1s) over time. If the probability of switching from 0 to 1 is high enough for an accumulation over the time frame of a lifespan, the sum of 1s can be used as the simplest predictor of age. The accumulation of DNA mutations could be seen as one example of this simplest case. Similarly, simulated stochastic changes in single-cell DNA methylation using an exponential decay approach starting with either 0 or 1 for all sites before applying stochastic changes, allowed for accurate predictions of the simulated age, in line with the regression-to-the-mean model, because each site starts at the extreme and can only diverge from it^33^.

In contrast to a multiplicative model, which shows a gradual slowdown of methylation change over time^33^, we modeled the stochastic variation accumulation in an additive manner, without a dependency of the random variation on the state of the system. We show that stochastic data-based clocks also predict chronological age and lifespan effects in transcriptome data of *C. elegans* and could measure the age deceleration resulting from reduced transcription drift through mianserin treatment^39^.

First-generation as well as second-generation DNA methylation aging clocks significantly correlate with the amount of stochastic variation in the data, suggesting that chronological and biological aging clocks are measuring stochastic variation. The prediction of all tested clocks plateaus after a certain amount of stochastic variation, possibly indicating an approach to site-specific equilibria. Cell-type composition was shown to change with age and to affect clock predictions^65,66^. Although this is an important aspect for the interpretation of clocks and the analysis of differentially methylated regions, correcting for cell-type composition did not change our results, and our DNA methylation simulations incorporating fixed or random maintenance rates cannot be confounded by a composition change over age. In line with this, age-related variably methylated positions are suggested to be not driven by variations in cell-type composition^29,67^. Publicly available clock predictions significantly correlate with the simulated age even if the same constant maintenance rate for all CpGs, or even random maintenance rates, are used. A cell-type corrected stochastic data-based clock maintains accurate predictions of independent cell-type corrected biological samples, underscoring that cell-type composition is not critical for the predictive power of stochastic variation accumulation. Although estimating *E*_m_ and *E*_d_ values is imperfect and likely cell-type dependent, our stochastic simulations are robust regardless of whether maintenance rates are estimated, randomly chosen or fixed at a universal value.

We replicated our results on data from the Mammalian Methylation Consortium^35^. Contrary to previous proposals that age-related CpG sites were not stochastic marks accrued with age^13–15^, our results show that a stochastic process and a single biological sample as the ground state are sufficient to: (1) build predictors significantly correlated with the relative age in various mammalian species; and (2) predict the age accelerating or decelerating effects of interventions such as GHRKO, calorie restriction or smoking.

Reprogramming via expression of the four transcription factors *Oct4* (also known as *Pou5f1*) *Sox2*, *Klf4* and *Myc* (OSKM) has been suggested to reverse cellular aging by resetting the DNA methylation landscape via de-differentiation^68^. Predictions with a stochastic data-based clock of a reprogramming time-course indeed follow the expected rejuvenation trajectory. Our work suggests that interventions (potentially even rejuvenation) could reduce and perhaps reverse stochastic variation.

That aging clocks strongly correlate with the amount of stochastic variation cautions the identification of causal effects. CpG sites that show faster stochastic variation accumulation are likely less efficiently maintained and less important for cell survival or homeostasis, making aging clock CpG sites unsuitable for the development of novel geroprotectors^10^. Indeed, many chronological aging clocks can be built from DNA methylation data and clock CpG sites might have limited value for understanding biology or anti-aging interventions^69^.

Stochastic data-based aging clocks demonstrate the compatibility of precise measures of the pace of aging with entropy-driven stochastic variations in biological processes such as age-associated damage accumulation. These results emphasize that a precise measure of aging pace does not require a programmed process, but is consistent with a stochastic nature of the molecular alterations. Although we show that accumulation of stochastic variation is sufficient to build aging clocks, the limitation of our study is that a deterministic aging trajectory could also be measured by a programmed clock. Thus, our results do not completely rule out the existence of deterministic processes. In certain species, deterministic processes regulate the aging process, as seen in variation in the monarch butterfly aging rate with migration routes^70^. Maintenance and repair mechanisms were selected during evolution for early, but not indefinite somatic maintenance, for instance the limitation of somatic DNA repair capacities by the DREAM complex in *C. elegans*^71^. Somatic proteostasis declines rapidly in nematodes becasue the heat shock response is repressed during reproduction onset via programmed *jmjd-3.1* reduction, which can be alleviated by removing the germline, consistent with the disposable soma theory^72^. The genetically programmed limitations of such maintenance and repair capacities could then result in age-dependent accumulation of stochastic damage.

Stochastic errors might start accumulating from conception, in line with the suggestion that aging starts from mid-embryonic development^73^. This might start a vicious spiral, because every additional error could disturb the intricate regulatory networks, including maintenance systems, thus allowing for more errors to be made^74^. It will be interesting to explore in how far a tightening of regulatory mechanisms could slow the aging process, consistent with EMS theory^26^.

We propose that in addition to methylation clocks, any set of biological measures, whether molecular or physiological, could in principle be used for building aging clocks, as long as the data have a range limit and experience accumulating stochastic variation. The sufficiency of stochasticity for building aging clocks unifies the exact determination of age and the reduced maintenance of homeostatic processes driving the aging process. Our analysis predicts that the level of such stochasticity sets the pace of aging. Reinstating regulatory tightness could therefore provide opportunities for aging decelerating therapies.

## Methods

### Bulk simulations

A ground state was generated with 2,000 (unless indicated otherwise) random features between 0 and 1. From this ground state 6 independent sets of 100 samples each (one sample per age from 1 to 100) were generated. Each of these 600 samples started from the same ground state with slight deviations; that is, each sample started with stochastic variation generated from *N*(*µ* = 0, *σ*^2^ = 0.01^2^) added to the ground state to simulate biological variation. To model age-dependent stochastic variation accumulation, random noise was generated from a normal distribution $N\left(\mu =0,{\sigma }^{2}\right)$ with random.randn() from Numpy v.1.18.5 (ref. ^75^). The standard deviation $\sigma$ used for generation of stochastic variation that is applied at each time-step is indicated in the figure legends. The simulated age of each sample defined how often stochastic variation generated from $N\left(\mu =0,{\sigma }^{2}\right)$ was independently added to the ground state. For example, for a sample with simulated age 2, stochastic variation would be added twice to the ground state. Stochastic variation addition was performed independently of all other samples, that is ground state +2× stochastic variation independently sampled from the normal distribution. A sample with simulated age 10 is acquired by taking the ground state and adding independently sampled, normal-distributed stochastic variation 10 times (Extended Data Fig. 1a). After stochastic variation addition values were kept between 0 and 1, by setting values larger than 1 to 1 and values smaller than 0 to 0 (except for the results in Extended Data Fig. 1c, where no limits where applied). To train a predictor of the simulated age we used 3 sets of 100 independent samples for training of an elastic net regression model using ElasticNetCV from sklearn v.0.23.1 (ref. ^76^) with the following parameter: l1_ratio = [0.1,0.2,0.3,0.4,0.5,0.6,0.7,0.8,0.9]. The remaining 3 sets of 100 independent samples were used as a hold-out validation dataset.

### Logit transform

Analysis undertaken with the logit transform was processed as follows. The ground state was first transformed with logit() from Scipy^77^. Stochastic variation was generated and applied as described above and added to the logit-transformed ground state. After stochastic variation addition, values were transformed back with the inverse-logit transform expit() from Scipy^77^.

### Human single-cell simulations

The ground state of single-cell simulations consists of 2,000 (unless indicated otherwise) randomly chosen CpG sites of the youngest sample in GSE41037 (ref. ^78^) (GSM1007467). For the clock starting from a fetal sample, a umbilical cord blood sample in GSE154915 (GSM4682890) was chosen. Each of the features (CpG sites) is a number between 0% and 100% and is used to generate 1,000 cells with binary values for each feature. A ground state value of 0.13 (13% methylated) generates 1,000 cells of which 130 are 1 (methylated) and 870 are 0 (unmethylated). One sample therefore consists of 2,000 (unless indicated otherwise) features each with 1,000 simulated cells with binary values of either 1 or 0. Note that our ground state is derived from bulk sequencing and not single-cell data, because single-cell omics come with large technical problems and drawbacks including the sparsity of sequencing coverage, which make it unfavorable as a starting point for our simulations^64^. Next, for each feature a methylation maintenance efficiency *E*_m_ and de novo methylation efficiency *E*_d_ were generated. As indicated in the figure legends, we either simulated data with a universal maintenance efficiency for all features, random efficiencies, or estimated *E*_m_ and *E*_d_ from empirical data. For the empirical maintenance estimation, we set the site-specific DNA methylation equilibrium as the value of the oldest sample in the dataset (GSM1007832)^78^, because DNA methylation trends toward the equilibrium over time^24,25^ and estimated *E*_m_ and *E*_d_ using the equation given by Pfeifer et al.^24^:

1 $${M}_{{\mathrm{eq}}}=\frac{{E}_{\mathrm{d}}}{1+{E}_{\mathrm{d}}-{E}_{\mathrm{m}}}$$

where *M*_eq_ is the equilibrium of the methylation state. Several groups have suggested a biological range for *E*_m_ and *E*_d_ values, with *E*_m_ being on average ~99.9% and *E*_d_ being ~5% (ref. ^24^), *E*_m_ being ~95% and for many sites >99% (ref. ^25^), or *E*_m_ being between 95% and 98% and *E*_d_ being maximally 23% (ref. ^79^). These limits guide our simulations, ensuring that both *E*_m_ and *E*_d_ are within biologically meaningful regions (95% < *E*_m_ ≤ 100% and 0% ≤ *E*_d_ < 23%). Note that the values inferred by those three publications only serve as an estimation of the biologically meaningful range for the methylation maintenance efficiency and the de novo methylation efficiency (95% < *E*_m_ ≤ 100% and 0% ≤ *E*_d_ < 23%). These three publications did not estimate site-specific values itself. Because of the nature of this empirical estimation either *E*_m_ or *E*_d_ is fixed, allowing the other to be estimated from data. Note that it is unlikely that all sites will have reached their equilibria with old age. This is therefore only a rough approximation of the site-specific equilibria, and multiple *E*_m_ and *E*_d_ values will regress to the same equilibrium over time (compare equation (1)). The lower the limit for *E*_m_, and respectively the higher the limit for *E*_d_, the higher the stochastic variation per time-step on average, because each site (feature) is potentially less well maintained, leading to a quicker regression to the equilibrium (perfect maintenance would be *E*_d_ = 0% and *E*_m_ = 100%). For example, CpG sites with *E*_m_ = 99% and *E*_d_ = 1% will regress toward 0.5 more slowly than CpG sites with *E*_m_ = 90% and *E*_d_ = 10%. Next, we randomly altered the state of every single-cell CpG site based on the respective *E*_m_ and *E*_d_ values for each time-step (for each time-step we flipped a coin with the probabilities *E*_m_ (to stay methylated) and *E*_d_ (to de novo methylate) for each CpG site in each cell). One hundred (unless indicated otherwise) age steps (stochastic variation applications) from 0 to 99 (unless indicated otherwise) were simulated. The simulations for GrimAge needed Illumina HumanMethylation450 BeadChip data and started from the youngest human blood sample in GSE40279 (GSM990528)^80^. Maintenance rates were estimated from the oldest sample (GSM989863). For training and validating a predictor, we again computed the average bulk methylation levels for each site and time point. The training and validation process of the elastic net regression is the same as described in Extended Data Fig. 1b.

### Cell-type correction

The cell-type composition was first estimated with EpiDISH^81^ with the parameter ref.m=centDHSbloodDMC.m and method=‘RPC’ in R-4.3. The estimated cell-type composition was subsequently used in a regression-based correction approach^82^. In brief, a linear model is fit for every CpG site using the cell-type composition values via lm(x~B+NK+CD4T+CD8T+Mono+Neutro+Eosino) to estimate the variance in the data that is predicted by the blood cell-type proportions. The remaining residuals depict the variance that is cell-type independent and can be added to the mean methylation value for each site to obtain the adjusted beta values^82^. In addition, we calculated a multivariate linear regression model of the form

$${\rm{Age}} \approx {\rm{PredictedAge}}+{\rm{CellTypeFractions}}$$

which gives *P* values for each of the variables and also whether the predicted age is significantly associated with the chronological age when also correcting for cell-type fractions.

### Public aging clocks

We downloaded the elastic net regression coefficients for Horvathʼs pan-tissue clock^26^, Vidal-Bralo’s blood aging clock^41^, Lin’s 99-CpG clock^42^, Weidner’s 3-CpG clock^43^ and Levine’s PhenoAge^40^ clock and applied them to the simulated data. The data were simulated as defined above, with the difference that we only used the clock-specific CpG sites as the features in the ground state, and we started the arbitrary simulated age at 16 (the age of the subject of the ground state sample). Stochastic variation was simulated either with a universal maintenance efficiency for all CpG sites or with empirically estimated maintenance rates as defined above. For GrimAge^44^ predictions we uploaded the simulated datasets to the webpage https://dnamage.genetics.ucla.edu/.

### Human stochastic data-based clock

The stochastic data-based clock was computed based on simulations described above. The scale and units of the simulated age are arbitrary because we do not know when or in which time-steps the noise increases, and are therefore different from the chronological age of biological samples. We found that a rescaling of the simulated age before training and testing the model is beneficial. First, we rescaled via min–max scaling the simulated age to be within 0 and 1, multiplied it by 400 and subtracted 120. Note that this transformation on the arbitrary time-steps will not interfere with the correlation analyses. For the correlation analyses, we excluded the youngest (GSM1007467, or GSM4682890; from which the ground state was sampled), and the oldest (GSM1007832; from which the maintenance efficiencies were estimated as described above) to not confound the correlation between the chronological age of samples in GSE41037 (ref. ^78^), and the predicted age. To train a predictor of the simulated age we used 1 set of 1 independent sample per age step from 1 to 73 for training of an elastic net regression model with ElasticNetCV from sklearn v.0.23.1 (ref. ^76^) with the following parameter: l1_ratio = [0.1,0.2,0.3,0.4,0.5,0.6,0.7,0.8,0.9], alphas = [1]. The clock was validated on 11,146 independent whole blood or peripheral blood leukocyte samples from the Illumina Infinium HumanMethylation450 BeadChip and the Illumina Infinium MethylationEPIC BeadChip (GSE84727, GSE87571, GSE80417, GSE40279, GSE87648, GSE42861, GSE50660, GSE106648, GSE179325, GSE210254, GSE210255, GSE72680, GSE147740, GSE55763, GSE117860).

### Pan-mammalian clocks

The pan-mammalian stochastic data-based clocks (clocks 1–4) are built on the youngest blood sample from *Tursiops truncatus* as the ground state (or stated otherwise) from the Illumina HorvathMammalianMethylChip40 BeadChip platform. Clock 1 used empirically estimated maintenance efficiency rates from the oldest sample of the same tissue and species as the ground state for all CpG sites of Lu’s pan-mammalian relative age clock. Clock 2 uses the same CpG sites, but nonempirically estimated a 99% maintenance rate for all sites (unless stated otherwise). Clock 3 is the same as clock 1 but utilizes all 37,554 CpG sites. Clock 4 is the same as clock 2 but utilizes all 37,554 CpG sites. To train a predictor of the simulated age we used 1 set of 1 independent sample per age step from 1 to 67 for training of an elastic net regression model with ElasticNetCV from sklearn v.0.23.1 (ref. ^76^) with the following parameter: l1_ratio = [0.01, 0.001], alphas = [1]. The predictor was trained to predict −log(−log(SimulatedAge/MaxAge)) as previously described^15^, where MaxAge is the number of age steps simulated (67). To get the relative age back, the predictions are transformed back via exp(−exp(−PredictedAge). Lu et al.^15^ used leave-one-fraction-out and leave-one-species-out cross-validation to get an unbiased estimate of the clock’s accuracy. Because the stochastic data-based clock needs only one biological sample as a ground state we directly applied the clock to all samples, thereby further reducing the risk of accuracy bias. To calculate the Pearson correlation of the predicted and relative age of species, only species with at least five samples (unless stated otherwise) were taken. Note that the species have distinct age ranges, which affects the Pearson correlation values. For validation of our stochastic data-based clocks on interventions with known lifespan effects for GHRKO, *Tet3*-knockout or CR mice, we calculated the adjusted false discovery rate and used the *t* value from a two-sided *t*-test for the color gradient (control versus experimental mice; a positive value indicates a younger predicted age in the experimental mice).

The statistics for the liver samples of the parabiosis dataset (GSE224361) and the slope difference of smoking individuals (GSE50660) were calculated with Python’s statsmodels.regression.linear_model.OLS and the following regression models:

Parabiosis (GSE224361):

$$\begin{array}{l}{\mathrm{PredictedAge}} \approx {\mathrm{ChronologicalAge}}+{\mathrm{HeterochronicParabiosis}}\\+\,{\mathrm{ChronologicalAge}}\times{\mathrm{HeterochronicParabiosis}}\end{array}$$

Where HeterochronicParabiosis is a binary variable indicating whether the parabiosis was heterochronic or isochronic.

Smoking (GSE50660):

$$\begin{array}{l}{\mathrm{PredictedAge}} \approx {\mathrm{ChronologicalAge}}+{\mathrm{ExSmoker}}+{\mathrm{CurrentSmoker}}\\+\,{\mathrm{ChronologicalAge}}\times{\mathrm{ExSmoker}}+{\mathrm{ChronologicalAge}}\times{\mathrm{CurrentSmoker}}\end{array}$$

Where ExSmoker and CurrentSmoker are binary variables indicating the smoking status of the sequenced individuals. The significant interaction term $\mathrm{{ChronologicalAge}\times{CurrentSmoker}}$ indicates a steeper slope (faster aging trajectory) and is shown as negative values in Fig. 5d. The smoking dataset and the reprogramming time-course dataset of human dermal fibroblasts (GSE54848)^49^ were generated with the Illumina Infinium HumanMethylation450 BeadChip array and was converted by the Array Converter Algorithm of the Mammalian Methylation Consortium before predicting the samples^15^.

### Gillespie algorithm

For the simulations we adapted the code from ref. ^83^. We modeled each CpG site with two different equations, one for the methylation and one for the demethylation. The probability of switching the state from one to the other was set to 0.1 for both equations. tmax was set to 5 and nrmax to 8,000. The arbitrary time-steps (of 0–5) were scaled to within the same range as the predicted age. Note that this does not affect the Pearson correlation results.

### Public RNA-seq processing

All 994 public RNA-seq samples were downloaded and processed in the same way. First, we preprocessed samples using Fastp v.0.20.0 (ref. ^84^) with the following parameters -g -x -q 30 -e 30. After preprocessing, the samples were mapped with Salmon v.1.1 (ref. ^85^) and the parameters –validateMappings –seqBias and additionally for paired-end samples, –gcBias. The decoy-aware index for Salmon was generated with the WS281 transcriptome build from Wormbase^86^. The results of Salmon were combined to the gene-level with tximport v.1.14.2 (ref. ^87^). Raw counts were log_10_-transformed after the addition of one pseudo-count, each sample was min–max normalized to bring each sample within the data range 0–1, and genes 0 in all 994 samples were filtered out. To binarize the data zeros were masked by NaN, the median was calculated; genes larger than the median were set to 1 and all other genes were set to 0 (ref. ^37^).

### Transcriptomic stochastic variation simulation

The ground state consists of all (unless indicated otherwise) gene counts (normalized as described above) of the biologically youngest sample (GSM2916344)^38^. From this ground state, ten independent samples for each time-step (from 1 to 16) were generated (based on the distribution that resulted in the best correlation with BiT age; Extended Data Fig. 2a) and used to train an elastic net regression as described above (see ‘Bulk simulations’). Note that the simulated age range is arbitrary, and the scale and unit are not directly comparable with the biological age. Similar to the epigenetic stochastic data-based clock, we found rescaling of the arbitrary simulated time-steps by two to be beneficial (we multiplied the simulated age by two before training and testing the data). The elastic net regression model was then used to predict the biological age of the 993 remaining *C. elegans* samples (excluding the youngest, which was used for the ground state). Biological age is calculated by temporal rescaling of the chronological age by the median lifespan. Briefly, we set a reference lifespan of a standard worm population to 15.5 days of adulthood and calculate a rescaling factor for every sample by dividing this reference lifespan by the median lifespan reported in the publication of the corresponding sample. This rescaling factor is multiplied with the chronological age of the sample^37^.

### Statistics and reproducibility

All indicated public data were used for validation, except for samples used as the ground state or to estimate maintenance rates as indicated. No statistical method was used to predetermine sample size. Stochastic variation accumulation simulations were done at least *N* = 3 times, as indicated in the figure legends, and can be reproduced with the public code. Data analyses were not performed blinded. The statistical tests used are indicated in the figure legends. Full statistics can be found in the Source Data. All data plots were done with Seaborn-0.11.0 (ref. ^88^) and Matplotlib-3.3.0 (ref. ^89^). Boxplots are shown with the center line depicting the median, the box limits showing the bottom and top quartiles, and the whiskers indicating the 1.5× interquartile range. Scatterplots showing a linear regression model fit are shown with a 95% confidence interval. Pearson correlations were computed with Scipy-1.5.1 stats.pearsonr function^77^ and two-sided tests. Effect sizes (Cohen’s *d* and Hedges’ *g*) for pair-wise comparisons were computed with Pingouin-0.3.6 compute_effsize function^90^.

### Reporting summary

Further information on research design is available in the Nature Portfolio Reporting Summary linked to this article.

### Supplementary information

Reporting Summary

Supplementary Code 1 Code to generate the simulations.

Supplementary Table 1 IDs and meta information for all 994 RNA-seq samples used.

### Source data

Source Data 1 Statistical source data for Figs. 1–6 and Extended Data Figs. 1 and 3–9.

## Data Availability

The human DNA methylation data is available at the National Center for Biotechnology Information Gene Expression Omnibus (GEO) database (accession code GSE84727, GSE87571, GSE80417, GSE40279, GSE87648, GSE42861, GSE50660, GSE106648, GSE179325, GSE210254, GSE210255, GSE72680, GSE147740, GSE55763, GSE117860, GSE41037, GSE54848, GSE223748 and GSE224361). The accession codes for all 994 public *Caenorhabditis* *elegans* RNA-seq samples can be found in Supplementary Table 1. The WS281 transcriptome version of *C. elegans* was downloaded from Wormbase^86^. Source data are provided with this paper.

## Code availability

The code for the simulations can be found in a supplementary file and at https://github.com/Meyer-DH/StochasticAgingClock. The BiT age clock code can be found at https://github.com/Meyer-DH/AgingClock. The Gillespie algorithm can be found at https://github.com/karinsasaki/gillespie-algorithm-python/blob/master/build_your_own_gillespie_solutions.ipynb. The ArrayConverterAlgorithm can be found at https://github.com/shorvath/MammalianMethylationConsortium/tree/main/UniversalPanMammalianClock/R_code/ArrayConverterAlgorithm.
