# Supplementary material for: Aging clocks based on accumulating stochastic variation
Source: Nat Aging. 2024 May 9;4(6):871–85. doi: 10.1038/s43587-024-00619-x (PMC11186771; doi:10.1038/s43587-024-00619-x)
Supplement: Supplementary file 1 — Reporting Summary [file 43587_2024_619_MOESM1_ESM.pdf]

nature portfolio

Corresponding author(s): David H. Meyer, Björn Schumacher

Last updated by author(s): 2024.02.22

Reporting Summary

Nature Portfolio wishes to improve the reproducibility of the work that we publish. This form provides structure for consistency and transparency in reporting. For further information on Nature Portfolio policies, see our Editorial Policies and the Editorial Policy Checklist.

Statistics

For all statistical analyses, confirm that the following items are present in the figure legend, table legend, main text, or Methods section.

n/a	Confirmed	
☐	☒	The exact sample size (*n*) for each experimental group/condition, given as a discrete number and unit of measurement
☐	☒	A statement on whether measurements were taken from distinct samples or whether the same sample was measured repeatedly
☐	☒	The statistical test(s) used AND whether they are one- or two-sided<br>*Only common tests should be described solely by name; describe more complex techniques in the Methods section.*
☐	☒	A description of all covariates tested
☐	☒	A description of any assumptions or corrections, such as tests of normality and adjustment for multiple comparisons
☐	☒	A full description of the statistical parameters including central tendency (e.g. means) or other basic estimates (e.g. regression coefficient) AND variation (e.g. standard deviation) or associated estimates of uncertainty (e.g. confidence intervals)
☐	☒	For null hypothesis testing, the test statistic (e.g. *F*, *t*, *r*) with confidence intervals, effect sizes, degrees of freedom and *P* value noted<br>*Give P values as exact values whenever suitable.*
☒	☐	For Bayesian analysis, information on the choice of priors and Markov chain Monte Carlo settings
☒	☐	For hierarchical and complex designs, identification of the appropriate level for tests and full reporting of outcomes
☐	☒	Estimates of effect sizes (e.g. Cohen's *d*, Pearson's *r*), indicating how they were calculated

Our web collection on statistics for biologists contains articles on many of the points above.

Software and code

Policy information about availability of computer code

Data collection	The raw RNA-seq files were downloaded from the GEO server with sratoolkit.3.0.1's prefetch, vdb-validate, and fasterq-dump functions.
Data analysis	The code for the simulations can be found at https://github.com/Meyer-DH/StochasticAgingClock. The BiT age clock code can be found at https://github.com/Meyer-DH/AgingClock.<br>The following Python packages were used:<br>Numpy v1.18.5<br>Pandas v1.0.5<br>Seaborn v0.11.0<br>Matplotlib v3.3.0<br>Scipy v1.5.1<br>Sklearn v0.23.1<br>Pingouin v0.3.6<br>Statsmodels v0.11.1<br>For cell-type composition estimation EpiDISH v2.16.0 in R v.4.3.0 was used.<br>The Gillespie algorithm can be found at https://github.com/karinsasaki/gillespie-algorithm-python/blob/master/build_your_own_gillespie_solutions.ipynb<br>The ArrayConverterAlgorithm can be found at https://github.com/shorvath/MammalianMethylationConsortium/tree/main/UniversalPanMammalianClock/R_code/ArrayConverterAlgorithm

For manuscripts utilizing custom algorithms or software that are central to the research but not yet described in published literature, software must be made available to editors and reviewers. We strongly encourage code deposition in a community repository (e.g. GitHub). See the Nature Portfolio guidelines for submitting code & software for further information.

Data

Policy information about availability of data

All manuscripts must include a data availability statement. This statement should provide the following information, where applicable:

- Accession codes, unique identifiers, or web links for publicly available datasets
- A description of any restrictions on data availability
- For clinical datasets or third party data, please ensure that the statement adheres to our policy

The human DNA methylation data is available at NCBI GEO (accession code GSE84727, GSE87571, GSE80417, GSE40279, GSE87648, GSE42861, GSE50660, GSE106648, GSE179325, GSE210254, GSE210255, GSE72680, GSE147740, GSE55763, GSE117860, GSE41037, GSE54848, GSE223748, and GSE224361). The accession codes for all 994 public C. elegans RNA-seq samples can be found in SupplementaryTable1.csv. The WS281 transcriptome version of C. elegans was downloaded from Wormbase.

Research involving human participants, their data, or biological material

Policy information about studies with human participants or human data. See also policy information about sex, gender (identity/presentation), and sexual orientation and race, ethnicity and racism.

Reporting on sex and gender	Not applicable
Reporting on race, ethnicity, or other socially relevant groupings	Not applicable
Population characteristics	Not applicable
Recruitment	Not applicable
Ethics oversight	Not applicable

Note that full information on the approval of the study protocol must also be provided in the manuscript.

Field-specific reporting

Please select the one below that is the best fit for your research. If you are not sure, read the appropriate sections before making your selection.

☒ Life sciences ☐ Behavioural & social sciences ☐ Ecological, evolutionary & environmental sciences

For a reference copy of the document with all sections, see nature.com/documents/nr-reporting-summary-flat.pdf

Life sciences study design

All studies must disclose on these points even when the disclosure is negative.

Sample size	~11k human blood DNA methylation samples were collected from public datasets. ~15k DNA methylation samples were collected from the Mammalian Methylation Consortium. The public C. elegans data consists of all RNA-seq sample for which we had information about the biological age via lifespan curves at hand. All simulations consist of at least 1 sample per integer age step and were repeated multiple times as indicated in the figure legends. Additionally we show in Figure 1F, 3D, Supplement Figure 1H, 2B,C, and 6B,C the effect of the feature size on the simulations. The indicated ~26k DNA methylation, and 993 RNA-seq validation data are downloaded from GEO, we did not control the sample sizes for these studies.
Data exclusions	Public samples were excluded if no chronological age was annotated.
Replication	All simulations are repeatable and can be directly reproduced with the attached code. Due to the random nature of the simulations, slight deviations are to be expected that will not interfere with any interpretation.
Randomization	All simulations are based on random numbers drawn from the described distributions.
Blinding	No group allocation was performed. Blinding of relevant meta information was not relevant since this is an observational study and all available data was used

Reporting for specific materials, systems and methods

We require information from authors about some types of materials, experimental systems and methods used in many studies. Here, indicate whether each material, system or method listed is relevant to your study. If you are not sure if a list item applies to your research, read the appropriate section before selecting a response.

Materials & experimental systems

n/a	Involved in the study
☒	☐ Antibodies
☒	☐ Eukaryotic cell lines
☒	☐ Palaeontology and archaeology
☒	☐ Animals and other organisms
☒	☐ Clinical data
☒	☐ Dual use research of concern
☒	☐ Plants

Methods

n/a	Involved in the study
☒	☐ ChIP-seq
☒	☐ Flow cytometry
☒	☐ MRI-based neuroimaging

Plants

Seed stocks	*Report on the source of all seed stocks or other plant material used. If applicable, state the seed stock centre and catalogue number. If plant specimens were collected from the field, describe the collection location, date and sampling procedures.*
Novel plant genotypes	*Describe the methods by which all novel plant genotypes were produced. This includes those generated by transgenic approaches, gene editing, chemical/radiation-based mutagenesis and hybridization. For transgenic lines, describe the transformation method, the number of independent lines analyzed and the generation upon which experiments were performed. For gene-edited lines, describe the editor used, the endogenous sequence targeted for editing, the targeting guide RNA sequence (if applicable) and how the editor was applied.*
Authentication	*Describe any authentication procedures for each seed stock used or novel genotype generated. Describe any experiments used to assess the effect of a mutation and, where applicable, how potential secondary effects (e.g. second site T-DNA insertions, mosiacism, off-target gene editing) were examined.*
